# An Improved Synthesis of Key Intermediate to the Formation of Selected Indolin-2-Ones Derivatives Incorporating Ultrasound and Deep Eutectic Solvent (DES) Blend of Techniques, for Some Biological Activities and Molecular Docking Studies †

**DOI:** 10.3390/molecules25051118

**Published:** 2020-03-02

**Authors:** Mohd Imran, Md. Afroz Bakht, Abida Khan, Md. Tauquir Alam, El Hassane Anouar, Mohammed B. Alshammari, Noushin Ajmal, Archana Vimal, Awanish Kumar, Yassine Riadi

**Affiliations:** 1 Department of Pharmaceutical Chemistry, Faculty of Pharmacy, Northern Border University, Rafha 91911, Saudi Arabia; Imran.pchem@gmail.com (M.I.); aqua_abkhan@yahoo.com (A.K.); tauquirpharm@gmail.com (M.T.A.); 2 Department of Chemistry, College of Science and Humanities in Al-Kharj, Prince Sattam Bin Abdulaziz University, Al-Kharj 11942, Saudi Arabia; anouarelhassane@yahoo.fr (E.H.A.); m.alshammari@psau.edu.sa (M.B.A.); 3 Department of Basic Sciences and Humanities, Pratap University, Jaipur 303104, Rajasthan, India; noush.biochem04@gmail.com; 4 Department of Biotechnology, National Institute of Technology Raipur C. G., Raipur 492010, India; avimal.phd2013.bt@nitrr.ac.in (A.V.); awanik.bt@nitrr.ac.in (A.K.); 5 Department of Pharmaceutical Chemistry, College of Pharmacy, Prince Sattam Bin Abdulaziz University, Al-Kharj 11942, Saudi Arabia; yassinriadi@yahoo.fr

**Keywords:** thiazole-indole, DES, ultrasound, anti-inflammatory, analgesic, ulcerogenic, lipid peroxidation, molecular docking, DFT

## Abstract

We have developed a new idea to synthesize a key intermediate molecule by utilizing deep eutectic solvent (DES) and ultrasound in a multistep reaction to ensure process cost-effectiveness. To confirm the stability of reagents with DES, electronic energies were calculated at the B3LYP/6-31+G(d,p) level of theory. DES stabilized the reagents mainly due to strong intermolecular hydrogen bonding. Key intermediate (3) and final compounds (**4a**–**n**) were synthesized in a higher yield of 95% and 80%–88%, respectively. Further, final compounds (**4a**–**n**) were assessed for their anti-inflammatory, analgesic, ulcerogenic, and lipid peroxidation. The compounds **4f**, **4g**, **4j**, **4l**, and **4m** showed good anti-inflammatory activity, while **4f**, **4i**, and **4n** exhibited very good analgesic activity as compared to the standard drug. The ulcerogenicity of selected compounds was far less than the indomethacin. The ligands had also shown a good docking score (**4f** = −6.859 kcal/mol and **4n** = −7.077 kcal/mol) as compared to control indomethacin (−6.109 kcal/mol) against the target protein COX-2. These derivatives have the potential to block this enzyme and can be used as NSAID. The state-of-art DFT theory was used to validate the lipid peroxidation mechanism of the active compounds which was in good agreement with the variations of BDEs and IP of the tested compounds.

## 1. Introduction

Nonsteroidal anti-inflammatory drugs (NSAIDs) are a profound application for the treatment of inflammatory diseases and pain. NSAIDs are the choice of treatment in various inflammation and pain-related problems such as osteoarthritis, rheumatoid arthritis, spondylitis, and gout [1,2,3]. A mechanism-based action of these drugs is exerted through the inhibition of cyclooxygenase type of enzymes, a principal enzyme that is used in the conversion of arachidonic acid to prostaglandin [4,5,6]. It has been reported that two forms of cyclooxygenase are involved in the pathogenesis of pain and inflammation, COX-1 and COX-2 [7,8]. However, their regulation and expression in the body are different [8,9], COX-1 is a known constitutive enzyme which helps in cytoprotection in the gastrointestinal tract (GI). The inhibition of COX-1 produces the undesired side effects of NSAIDs, for example, gastrointestinal toxicity because of their ulcerogenic effects. The COX-2 is an inducible enzyme that works through the mediation of the selective inflammatory signal and the therapeutic anti-inflammatory action of NSAIDs is produced by the inhibition of COX-2 [10,11,12,13,14]. Based on this observation, many selective COX-2 inhibitors such as celecoxib, rofecoxib, and valdecoxib emerged as relatively safe NSAID’S together with improved gastric problems. However, the reporting of the cardiovascular side effects, for example, increased risk of myocardial infarction, stroke, heart failure, and hypertension caused the withdrawal of many COX-2 inhibitors from the market [15]. This encouraged research professionals to develop newer chemical entities as anti-inflammatory agents with minimal side effects.

Indole ring and its derivatives have emerged as privileged pharmacophores representing more than thousands of natural isolates with known biological and pharmaceutical activities such as anti-inflammatory, analgesic [16,17,18,19], antimicrobial [20], antitumor [21], and anticonvulsant [22]. This ring is also a vital part of indomethacin, which is currently marketed as NSAIDs. However, the gastric safety profile of indomethacin is not promising and it produces gastrointestinal toxicity because of its ulcerogenic effects. In recent times, research reports highlighting the usefulness of the development of new coumarinylthiazoles as an anti-inflammatory and analgesic agents have also been published [23,24,25]. Thiazole and indole type of moieties were reported to synthesize by utilizing harsh chemicals/solvents which causes environmental pollution, as well as raise the risk of health issues [26,27]. An alternative to such solvents such as deep eutectic solvent (DES) is the most valuable choice for varieties of organic transformations [28,29]. DES is usually a mixture of compounds having melting points less than their mixing components. The most versatile DES was prepared from choline chloride and some hydrogen bond donor (urea, glycerol) [29]. Depression in the melting point of DES is associated with the molecular interaction of choline chloride and hydrogen bond donor part [29].

The immense application of ultrasound has been highlighted recently in organic and material science [30,31]. Ultrasound increased the rate of reaction by acoustic cavitation phenomena generated as a result of initiation, growth, and collapse of bubbles during the course of reactions.

Keeping these things and with extended work [32,33,34,35] of our group to the development of new chemical templates in order to discover novel NSAIDs, authors planned to synthesize some molecules with a low budget and utilizing deep eutectic solvent and ultrasound technique to fulfill green chemistry approach.

## 2. Results and Discussion

### 2.1. Chemistry

**Scheme 1:**
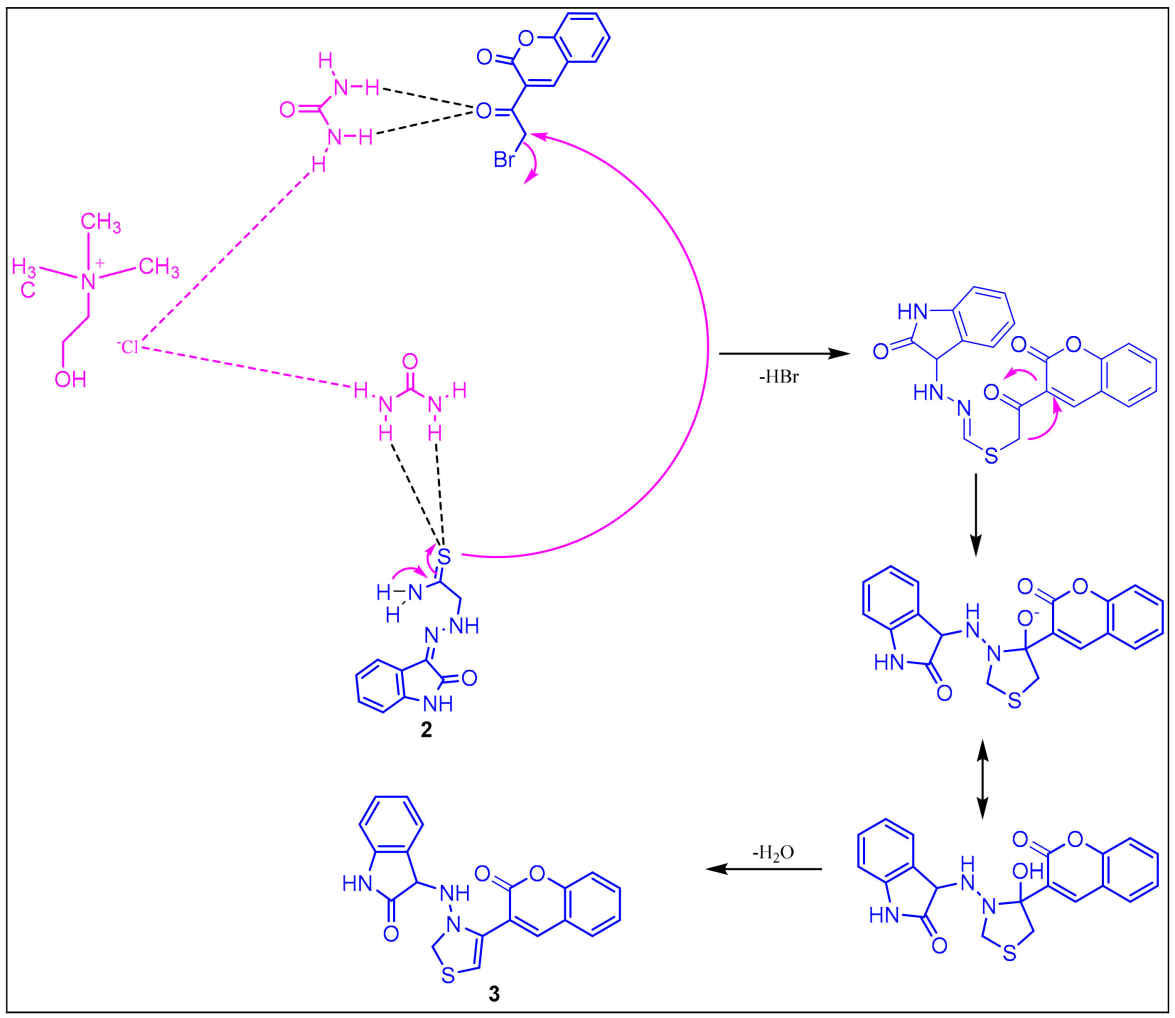
The proposed mechanism involved in the formation of key intermediate, 3-(2-(4-(2-oxochroman-3-yl) thiazol-2-yl) hydrazono) indolin-2-one (3) using deep eutectic solvent (DES).

1-(Substitutedphenylaminomethyl)-3-(2-(4-(2-oxochroman-3-yl)thiazol-2-yl)hydrazono)indolin-2-ones were synthesized by treating 3-(2-(4-(2-oxochroman-3-yl)thiazol-2-yl)hydrazono) indolin-2-one (**3**) with substituted aromatic amines and formaldehyde in ethylene glycol as depicted in Scheme 1. Prepared compounds were elucidated by FT-IR, ^1^H-NMR, ^13^C-NMR, mass and elemental analysis. In general, absorption bands due to two -NH groups appeared in the IR spectra at around 3200 cm^−1^. Other bands due to -C=N and two -C=O functional groups were found at around 1600 and 1700, respectively. In the ^1^H-NMR spectra, two -NH peaks appeared at around 9 and 10 ppm. The lower value provides information as a singlet due to -NH attached as -CH_2_NH with indolinone nitrogen as a characteristic peak. Value at δ 5 ppm confirms the presence of -CH_2_ which is another important peak for identification. Further, characteristics peak of -CH_2_ of -CH_2_NH was confirmed by ^13^C-NMR around δ 69 ppm. 

The characterization data of all the synthesized compounds are provided below.

*2-(2-Oxoindolin-3-ylidene)hydrazine carbothioamide (**2**)*: m.p.: 222–224 °C; %Yield: 72; IR (KBr) cm^−1^: 3413, 3352, and 3216 (N-H), 1693 (C=O).^1^H-NMR (CDCl_3_, DMSO-*d*_6_) ppm: 6.72 (s, 1H, NH), 6.92 (d, *J* = 12 Hz, 1H, Ar-H), 7.03 (t, *J* = 8 Hz, 1H, Ar-H), 7.34 (t, *J* = 8 Hz, 1H, Ar-H), 8.04 (d, *J* = 12 Hz 1H, Ar-H), 9.99 (s, 1H, NH), 10.55 (s, 2H, NH); elemental analysis: Calcd. for (C_9_H_8_N_4_OS), found % (calculated %): C, 49.07 (49.08); H, 3.65 (3.66); N, 25.43 (25.44).

*3-(2-(4-(2-Oxo-2H-chromen-3-yl)-4,5-dihydrothiazol-2-yl)hydrazono)indolin-2-one(**3**)*: m.p.: 240–242 °C; %Yield: 95; IR (KBr) cm^−1^: 1692 and 1703 (C=O), 3315 and 3253 (N-H), 1612 (C=N), 1543 (C=C).1H-NMR (CDCl3, DMSO-*d*_6_) ppm: 7.03 (t, *J* = 8 Hz, 1H, Ar-H), 7.39 (m, 8H, Ar-H, NH), 8.28 (s, 2H, Ar-H), 10.25 (s, 1H, -NH=N-); elemental analysis: Calcd. for (C_20_H_12_N_4_O_3_S), found % (calculated %): C, 61.84 (61.85); H, 3.10 (3.11); N, 14.42 (14.43).

*3-{[4-(2-Oxo-2H-chromen-3-yl)-thiazol-2-yl]-hydrazono}-1-phenylaminomethyl-1,3-dihydro-indol-2-one (**4a**)*: m.p.: 245–247 °C; %Yield: 85; IR (KBr) cm^−1^: 1683 and 1710 (C=O), 3309 and 3251 (N-H), 1613 (C=N), 1546 (C=C); ^1^H-NMR (CDCl_3_, DMSO-*d*_6_) ppm: 5.13 (s, 2H, CH_2_), 7.44–8.10 (m, 13H, Ar-H), 9.35 (s, 1H, NH), 10.53 (s, 1H, NH); ^13^C-NMR (125 MHz, DMSO-*d*_6_); 171.0 (C=N, thiazolidine),159 and 162 (2CO), 156 (1C, C=N), 143.4, 140.9, 139.07, 139.0, 138.6, 131.0, 129.8, 129.5, 127.8, 126.8, 125.5, 124.3, 123.4, 121.2, 117.1, 112.4, (Ar-C), 69.3 (1C, CH2); elemental analysis: Calcd. for (C_27_H_19_N_5_O_3_S), found % (calculated %): C, 65.70 (65.71); H, 3.87 (3.88); N, 14.18 (14.19). Mass (*m*/*z*): 493 (M+, C_27_H_19_N_5_O_3_S), 200 (C_11_H_6_NOS), 175 (C_10_H_7_OS), 168 (C_12_H_10_N), 159 (C_8_H_7_N_4_), 132 (100%, C_7_H_6_N_3_), 106 (C_7_H_8_N).

*1-[(4-Fluoro-phenylamino)-methyl]-3-{[4-(2-oxo-2H-chromen-3-yl)-thiazol-2-yl]-hydrazono}-1,3-dihydro-indol-2-one (**4b**)*: m.p.: 242–244 °C; %Yield: 82; IR (KBr) cm^−1^: 1683 and 1704 (C=O), 3312 and 3264 (N-H), 1613 (C=N), 1543 (C=C); ^1^H-NMR (CDCl_3_, DMSO-*d*_6_) ppm: 5.10 (s, 2H, CH_2_), 7.42 (m, 14H, Ar-H), 9.15 (s, 1H, NH), 10.55 (s, 1H, NH); 13C-NMR (125 MHz, DMSO-*d*_6_); 172.0 (C=N, thiazolidine), 160 and 161 (2CO), 155 (1C, C=N), 145.2, 143.1, 140.2, 139.0, 138.4, 132.5, 130.7, 129.3, 128.9, 127.6, 125.3, 124.4, 122.2, 118.3, 114.3, (Ar-C), 68.9 (1C, CH_2_); MS (*m*/*z*): 511 (M^+^), 513 (M^+^ + 2); elemental analysis: Calcd. for (C_27_H_18_N_5_O_3_SF), found % (Calculated %): C, 63.38 (63.40); H, 3.55 (3.55); N, 13.68 (13.69).

*1-[(4-Chloro-phenylamino)-methyl]-3-{[4-(2-oxo-2H-chromen-3-yl)-thiazol-2-yl]-hydrazono}-1,3-dihydro-indol-2-one (**4c**)*: m.p.: 233–235 °C; %Yield: 85; IR (KBr) cm^−1^: 1689 and 1705 (C=O), 3309 and 3251 (N-H), 1613 (C=N), 1544 (C=C); 1H-NMR (CDCl_3_, DMSO-*d*_6_) ppm: 5.14 (s, 2H, CH_2_), 7.39 (m, 14H, Ar-H), 9.25 (s, 1H, NH), 10.50 (s, 1H, NH); ^13^C-NMR (125 MHz, DMSO-*d*_6_); 170.0 (C=N, thiazolidine), 161 and 162 (2C, C=O), 157 (1C, C=N), 146.1, 143.1, 140.3, 139.1, 138.6, 133.5, 131.7, 129.2, 128.4, 127.7, 126.3, 125.2, 123.1, 117.9, 112.8, (Ar-C), 68.6 (1C, CH_2_); MS (*m*/*z*): 528 (M^+^), 530 (M^+^ + 2); elemental analysis: Calcd. for (C_27_H_18_N_5_O_3_SCl), found % (calculated %): C, 61.41 (61.42); H, 3.44 (3.44); N, 13.25 (13.26).

*1-[(4-Bromo-phenylamino)-methyl]-3-{[4-(2-oxo-2H-chromen-3-yl)-thiazol-2-yl]-hydrazono}-1,3-dihydro-indol-2-one (**4d**)*: m.p.:241–243 °C; %Yield: 80; IR (KBr) cm^−1^: 1685 and 1704 (C=O), 3313 and 3251 (N-H), 1611 (C=N), 1547 (C=C); ^1^H-NMR (CDCl_3_, DMSO-*d*_6_) ppm: 5.14 (s, 2H, CH_2_), 7.40 (m, 14H, Ar-H), 9.30 (s, 1H, NH), 10.51 (s, 1H, NH); ^13^C-NMR (125 MHz, DMSO-*d*_6_); 171.0 (C=N, thiazolidine), 162 and 163 (2C, C=O), 156 (1C, C=N), 145.5, 143.6, 140.3, 139.8, 138.3, 133.6, 131.4, 129.7, 128.1, 127.7, 126.9, 123.6, 118.9, 112.9, (Ar-C), 69.3 (1C, CH_2_); MS (*m*/*z*): 572 (M^+^), 574 (M^+^ + 2); elemental analysis: Calcd. for (C_27_H_18_N_5_O_3_SBr), found % (calculated %): C, 56.64 (56.65); H, 3.16 (3.17); N, 12.22 (12.23).

*1-[(2-Nitro-phenylamino)-methyl]-3-{[4-(2-oxo-2H-chromen-3-yl)-thiazol-2-yl]-hydrazono}-1,3-dihydro-indol-2-one (**4e**)*: m.p.: 244–246 °C; %Yield: 85; IR (KBr) cm^−1^: 1684 and 1705 (C=O), 3310 and 3255 (N-H), 1613 (C=N), 1543 (C=C); ^1^H-NMR (CDCl_3_, DMSO-*d*_6_) ppm: 5.07 (s, 2H, CH_2_), 7.42 (m, 14H, Ar-H), 9.15 (s, 1H, NH), 10.48 (s, 1H, NH); ^13^C-NMR (125 MHz, DMSO-*d*_6_); 170.0 (C=N, thiazolidine), 163 and 164 (2CO), 157 (1C, C=N), 146.4, 143.8, 141.6, 140.4, 139.7, 135.6, 133.4, 130.7, 128.5, 127.5, 126.4, 124.6, 118.6, 112.3, (Ar-C), 70.1 (1C, CH2); MS (*m*/*z*): 538 (M^+^), 540 (M^+^ + 2); elemental analysis: Calcd. for (C_27_H_18_N_6_O_5_S), found % (calculated %): C, 60.21 (60.22); H, 3.36 (3.37); N, 15.60 (15.61).

*1-[(2-chloro-phenylamino)-methyl]-3-{[4-(2-oxo-2H-chromen-3-yl)-thiazol-2-yl]-hydrazono}-1,3-dihydro-indol-2-one (**4f**)*: m.p.: 239–241 °C; %Yield: 83; IR (KBr) cm^−1^: 1689 and 1707 (C=O), 3311 and 3252 (N-H), 1613 (C=N), 1545 (C=C); ^1^H-NMR (CDCl_3_, DMSO-*d*_6_) ppm: 5.10 (s, 2H, CH_2_), 7.36 (m, 14H, Ar-H), 9.36 (s, 1H, NH), 10.55 (s, 1H, NH); ^13^C-NMR (125 MHz, DMSO-*d*_6_); 172.0 (C=N, thiazolidine), 161 and 162 (2CO), 157 (1C, C=N), 146.5, 143.9, 140.8, 140.1, 138.7, 135.4, 133.7, 129.7, 128.8, 127.6, 126.3, 124.7, 119.3, 114.1, (Ar-C), 68.3 (1C, CH_2_); elemental analysis: Calcd. for (C_27_H_18_N_5_O_3_SCl), found % (calculated %): C, 61.41 (61.42); H, 3.43 (3.44); N, 13.25 (13.26). Mass (*m*/*z*): 527 (M^+^, C_27_H_18_N_5_O_3_SCl), 528 (M^+^ + 1), 202 (C_12_H_9_NCl), 175 (C_10_H_7_OS), 132 (100%, C_7_H_6_N_3_), 111 (C_6_H_4_Cl), 59 (C_2_H_3_S).

*1-[(2,4-Dinitro-phenylamino)-methyl]-3-{[4-(2-oxo-2H-chromen-3-yl)-thiazol-2-yl]-hydrazono}-1,3-dihydro-indol-2-one (**4g**)*: m.p.: 247–249 °C; %Yield: 80; IR (KBr) cm^−1^: 1686 and 1704 (C=O), 3292 and 3252 (N-H), 1612 (C=N), 1544 (C=C); ^1^H-NMR (CDCl_3_, DMSO-*d*_6_) ppm: 5.15 (s, 2H, CH_2_), 7.45 (m, 13H, Ar-H), 9.33 (s, 1H, NH), 10.51 (s, 1H, NH); ^13^C-NMR (125 MHz, DMSO-*d*_6_); 169.0 (C=N, thiazolidine), 159 and 161 (2CO), 155 (1C, C=N), 145.7, 142.5, 140.2, 139.1, 137.4, 135.7, 133.5, 129.6, 128.6, 127.3, 124.7, 118.9, 112.3, (Ar-C), 69.7 (1C, CH_2_); MS (*m*/*z*): 583 (M^+^), 585 (M^+^ + 2); elemental analysis: Calcd. for (C_27_H_17_N_7_O_7_S), found % (calculated %): C, 55.56 (55.57); H, 2.94 (2.94); N, 16.79 (16.80).

*3-{[4-(2-Oxo-2H-chromen-3-yl)-thiazol-2-yl]-hydrazono}-1-([1,2,4]triazol-4-ylaminomethyl)-1,3-dihydro-indol-2-one (**4h**)*: m.p.: 236–238 °C; %Yield: 80; IR (KBr) cm^−1^: 1685 and 1706 (C=O), 3253 and 3279 (N-H), 1613 (C=N), 1543 (C=C); ^1^H-NMR (CDCl_3_, DMSO-*d*_6_) ppm: 5.17 (s, 2H, CH_2_), 7.48 (m, 12H, Ar-H), 9.42 (s, 1H, NH), 10.47 (s, 1H, NH); ^13^C-NMR (125 MHz, DMSO-*d*_6_); 170.0 (C=N, thiazolidine), 161 and 161 (2CO), 157 (1C, C=N), 144.8, 140.2, 137.4, 135.9, 132.4, 129.4, 128.7, 127.5, 124.3, 116.9, 112.3, (Ar-C), 70.2 (1C, CH_2_); MS (*m*/*z*): 484 (M^+^), 486 (M^+^ + 2); elemental analysis: Calcd. for (C_23_H_16_N_8_O_3_S), found % (calculated %): C, 57.01 (57.02); H, 3.32 (3.33); N, 23.12 (23.13).

*1-[(3-Chloro-4-fluoro-phenylamino)-methyl]-3-{[4-(2-oxo-2H-chromen-3-yl)-thiazol-2-yl]-hydrazono}-1,3-dihydro-indol-2-one (**4i**)*: m.p.: 246–248 °C; %Yield: 85; IR (KBr) cm^−1^: 1684 and 1703 (C=O), 3240 and 3273 (N-H), 1612 (C=N), 1544 (C=C); ^1^H-NMR (CDCl_3_, DMSO-*d*_6_) ppm: 5.05 (s, 2H, CH_2_), 7.41 (m, 13H, Ar-H), 9.31 (s, 1H, NH), 10.55 (s, 1H, NH); ^13^C-NMR (125 MHz, DMSO-*d*_6_); 171.0 (C=N, thiazolidine), 161 and 163 (2CO), 156 (1C, C=N), 146.9, 143.4, 140.6, 139.2, 137.6, 135.7, 133.8, 129.3, 128.3, 127.6, 124.2, 117.4, 112.3, (Ar-C), 68.6 (1C, CH_2_); MS (*m*/*z*): 546 (M^+^), 548 (M^+^ + 2); elemental analysis: Calcd. for (C_27_H_17_N_5_O_3_SClF), found % (calculated %): C, 59.38 (59.40); H, 3.14 (3.14); N, 12.82 (12.83).

*3-{[4-(2-Oxo-2H-chromen-3-yl)-thiazol-2-yl]-hydrazono}-1-(pyridine-4-ylaminomethyl)-1,3-dihydro-indol-2-one (**4j**)*: m.p.: 237–239 °C; %Yield: 88; IR (KBr) cm^−1^: 1687 and 1705 (C=O), 3244 and 3268 (N-H), 1613 (C=N), 1545 (C=C); ^1^H-NMR (CDCl_3_, DMSO-*d*6) ppm: 5.17 (s, 2H, CH_2_), 7.45 (m, 14H, Ar-H), 9.38 (s, 1H, NH), 10.57 (s, 1H, NH); ^13^C-NMR (125 MHz, DMSO-*d*_6_); 172.0 (C=N, thiazolidine), 161 and 163 (2CO), 156 (1C, C=N), 144.8, 143.5, 140.4, 139.0, 138.3, 132.5, 130.9, 129.3, 128.8, 127.6, 125.1, 124.7, 122.9, 116.9, 112.6, (Ar-C), 68.3 (1C, CH_2_); MS (*m*/*z*): 594 (M^+^), 596 (M^+^ + 2); elemental analysis: Calcd. for (C_26_H_18_N_6_O_3_S), found % (Calculated %): C, 63.14 (63.15); H, 3.66 (3.67); N, 16.98 (16.99).

*3-{[4-(2-Oxo-2H-chromen-3-yl)-thiazol-2-yl]-hydrazono}-1-(pyridine-3-ylaminomethyl)-1,3-dihydro-indol-2-one (**4k**)*: m.p.: 231–233 °C; %Yield: 85; IR (KBr) cm^−1^: 1686 and 1706 (C=O), 3251 and 3277 (N-H), 1612 (C=N), 1543 (C=C); ^1^H-NMR (CDCl_3_, DMSO-*d*6) ppm: 5.15 (s, 2H, CH_2_), 7.47 (m, 14H, Ar-H), 9.35 (s, 1H, NH), 10.55 (s, 1H, NH); ^13^C-NMR (125 MHz, DMSO-*d*_6_); 172.0 (C=N, thiazolidine), 161 and 162 (2CO), 156 (1C, C=N), 144.9, 143.6, 140.2, 139.0, 138.5, 132.3, 130.7, 129.5, 128.7, 127.3, 125.5, 124.9, 122.8, 116.6, 112.4, (Ar-C), 68.3 (1C, CH_2_); MS (*m*/*z*): 594 (M^+^), 596 (M^+^ + 2); elemental analysis: Calcd. for(C_26_H_18_N_6_O_3_S), found % (calculated %): C, 63.14 (63.15); H, 3.66 (3.67); N, 16.98 (16.99).

*1-[(4-Nitro-phenylamino)-methyl]-3-{[4-(2-oxo-2H-chromen-3-yl)-thiazol-2-yl]-hydrazono}-1,3-dihydro-indol-2-one (**4l**)*: m.p.: 241–243 °C; %Yield: 90; IR (KBr) cm^−1^: 1684 and 1702 (C=O), 3255 and 3278 (N-H), 1612 (C=N), 1544 (C=C); ^1^H-NMR (CDCl_3_, DMSO-*d*_6_) ppm: 5.18 (s, 2H, CH_2_), 7.46 (m, 14H, Ar-H), 9.32 (s, 1H, NH), 10.54 (s, 1H, NH); ^13^C-NMR (125 MHz, DMSO-*d*_6_); 170.0 (C=N, thiazolidine), 163 and 164 (2CO), 158 (1C, C=N), 146.6, 143.7, 141.9, 140.5, 139.7, 135.2, 133.5, 130.1, 128.2, 127.7, 126.1, 124.3, 116.9, 112.3, (Ar-C), 70.2 (1C, CH_2_); MS (*m*/*z*): 538 (M^+^), 540 (M^+^ + 2); elemental analysis: Calcd. for (C_27_H_18_N_6_O_5_S), found % (calculated %): C, 60.21 (60.22); H, 3.36 (3.37); N, 15.60 (15.61).

*3-{[4-(2-Oxo-2H-chromen-3-yl)-thiazol-2-yl]-hydrazono}-1-(p-tolylamino-methyl)-1,3-dihydro-indol-2-one (**4m**)*: m.p.: 244–246 °C; %Yield: 84; IR (KBr) cm^−1^: 1685 and 1706 (C=O), 3252 and 3273 (N-H), 1608 (C=N), 1544 (C=C); ^1^H-NMR (CDCl_3_, DMSO-*d*_6_) ppm: 2.23 (s, 3H, CH_3_), 5.11 (s, 2H, CH2), 7.41 (m, 14H, Ar-H), 9.31 (s, 1H, NH), 10.48 (s, 1H, NH); ^13^C-NMR (125 MHz, DMSO-*d*_6_); 172.0 (C=N, thiazolidine), 160 and 163 (2CO), 156 (1C, C=N), 146.8, 144.2, 142.4, 140.6, 139.9, 135.3, 133.8, 130.5, 128.8, 127.1, 126.3, 124.8, 117.4, 114.1, (Ar-C), 70.3 (1C, CH_2_); MS (*m*/*z*): 507 (M^+^), 509 (M^+^ + 2); elemental analysis: Calcd. for (C_28_H_21_N_5_O_3_S), found % (calculated %): C, 66.25 (66.26); H, 4.16 (4.17); N, 13.79 (13.80).

*3-{[4-(2-Oxo-2H-chromen-3-yl)-thiazol-2-yl]-hydrazono}-1-(o-tolylamino-methyl)-1,3-dihydro-indol-2-one (**4n**)*:m.p.: 237–239 °C; %Yield: 86; IR (KBr) cm^−1^: 1686 and 1703 (C=O), 3251 and 3282 (N-H), 1612 (C=N), 1543 (C=C); 1H-NMR (CDCl_3_, DMSO-*d*_6_) ppm: 2.21 (s, 3H, CH_3_), 5.13 (s, 2H, CH2), 7.43 (m, 14H, Ar-H), 9.34 (s, 1H, NH), 10.53 (s, 1H, NH); ^13^C-NMR (125 MHz, DMSO-*d*_6_); 171.0 (C=N, thiazolidine), 161 and 162 (2CO), 157 (1C, C=N), 145.9, 144.2, 142.6, 140.3, 139.2, 135.6, 133.5, 130.2, 128.9, 127.6, 126.1, 124.9, 118.2, 112.3, (Ar-C), 68.3 (1C, CH_2_); elemental analysis: Calcd. for (C_28_H_21_N_5_O_3_S), found % (calculated %): C, 66.25 (66.26); H, 4.16 (4.17); N, 13.80 (13.80); Mass (*m*/*z*): 507 (M^+^, C_28_H_21_N_5_O_3_S), 387 (C_20_H_11_N_4_O_3_S), 200 (C_11_H_6_NOS), 132 (100%, C_7_H_6_N_3_), 120 (C_8_H_10_N), 90 (C_6_H_4_N), 59 (C_2_H_3_S).

#### 2.1.1. Significance of DES and Ultrasound Blend of Techniques to the Synthesis of Key Intermediate 3-(2-(4-(2-Oxochroman-3-Yl) thiazol-2-yl) Hydrazono) Indolin-2-One

To develop the efficient method as compared to conventional, we have conducted the synthesis of key intermediate (3) utilizing biocompatible deep eutectic solvent (DES) and ultrasound blend of technique. As a result of combined use of DES and ultrasound, an increase in % yield of key intermediate as high as 95% has been found with the expense of 1 h only. Whereas, a similar type of organic transformation using dioxane and another organic solvent together with conventional heating were reported to have % yield around 44%–68% in 3–4 h [36,37,38]. Further, we have also found 80%–88% of all final compounds (**4a**–**n**) utilizing ultrasound as a source of heating. Some of our earlier work and other related literature also mentioned the significance DES and ultrasound technology as an energy saving process [29,39,40] which is certainly a good favor of our present work.

#### 2.1.2. Plausible Mechanism Involved in the Formation of Key Intermediate, 3-(2-(4-(2-Oxochroman-3-Yl) Thiazol-2-yl) Hydrazono) Indolin-2-One

The exact mechanism of formation of the desired intermediate compound is not yet clear. However, it was suggested by some researchers that the urea part of DES (choline chloride: urea, 1:2) catalyzes the reaction by making hydrogen bond. Thus, urea in a deep eutectic solvent was involved in stabilizing the acetyl moiety of 3-bromoacetylcoumarin via hydrogen bonding, which was further attacked by amide functional group of hydrazine thioamide to form key intermediate, 3-(2-(4-(2-oxochroman-3-yl) thiazol-2-yl) hydrazono) indolin-2-one through cyclization and dehydration process (Scheme 1).

**Figure 1:**
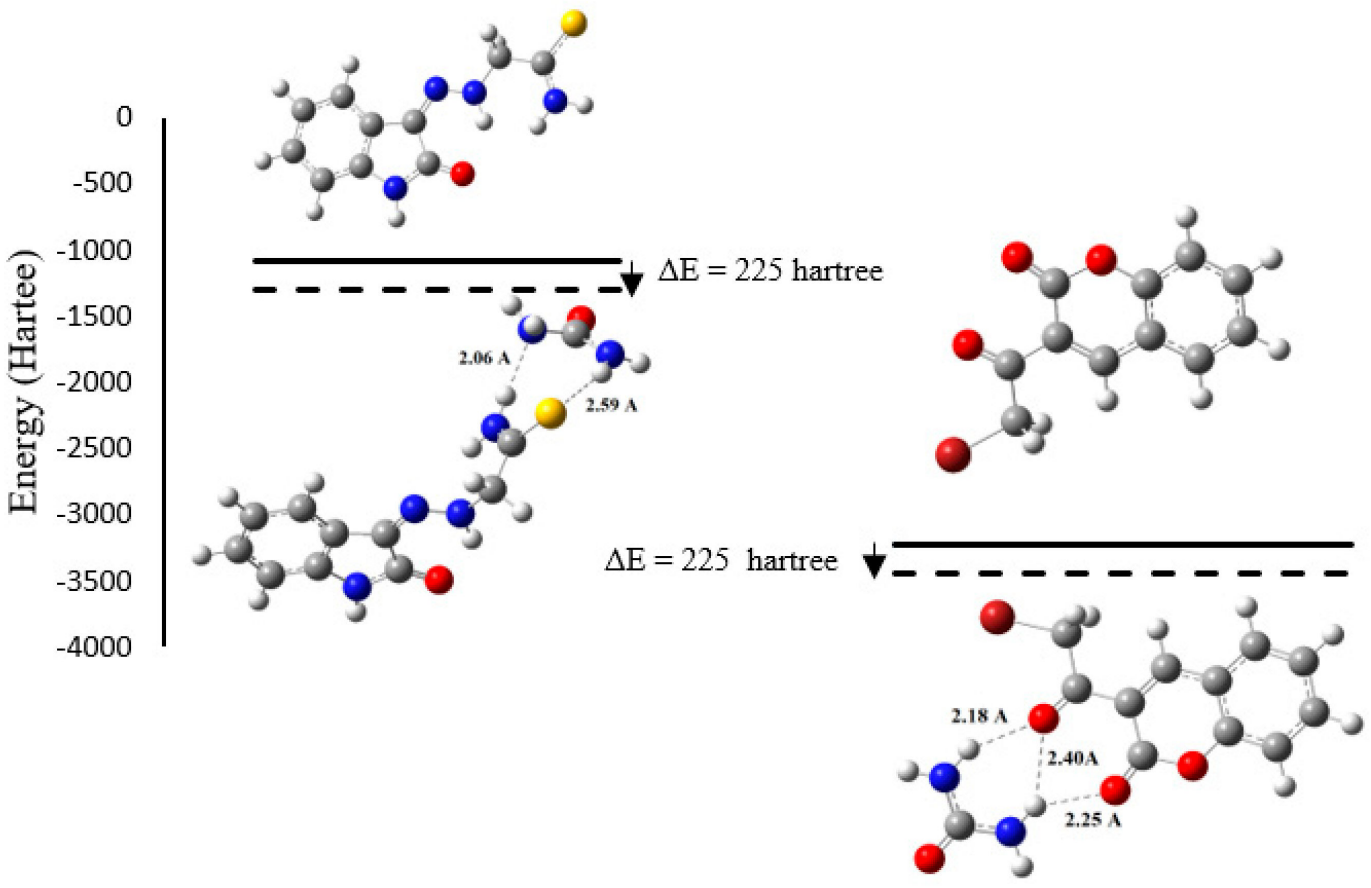
Energetic diagram of starting materials in the presence and absence of DES (dotted lines).

To emphasize the role of DES in the stability of starting reagents, the electronic energies of the latter were calculated at the B3LYP/6-31+G(d,p) level of theory in the presence and absence of DES (Figure 1). The presence of DES stabilizes both reagents by 225 hartree (Figure 1). The stability of the starting material in the presence of DES is mainly referred to the formation of strong intermolecular hydrogen bonding between DES and the starting reagents (Figure 1).

Moreover, ultrasound also played a significant role in the formation of the desired compound. Under the influence of sonic waves inside the reaction vessel, there was the formation of microscopic bubbles, as a result of high temperature and pressure [28,29,30,31]. These tiny microscopic bubbles also help in the cyclization process.

### 2.2. Biology

#### 2.2.1. Anti-Inflammatory Activity

The anti-inflammatory activity of the synthesized compounds (**4a**–**n**) was evaluated by the carrageenan-induced paw edema method. An oral dose of 10 mg/kg was used for compounds and compared with the standard. Anti-inflammatory activity was accessed through percentage inhibition after 2 and 4 h (Table 1).

Anti-inflammatory activity in terms of percentage inhibition for the test compounds is ranging from 5.57% to 80.94% (Table 1), whereas the standard drug showed 82.05% after 4 h. Compounds **4f** (72.42%), **4g** (77.94%), **4j** (77.90%), **4k** (70.75%), **4l** (80.94%), and **4m** (78.42%) showed comparable results against the standard drug.

The structure of 1-(substituted phenyl amino methyl)-3-(2-(4-(2-oxochroman-3-yl) thiazol-2-yl) hydrazono) indolin-2-one derivatives revealed that the compound **4l** (Ar = 4-nitrophenyl) exhibited the highest anti-inflammatory activity. Other compounds of the series, namely, **4f** (Ar = 2-chlorophenyl), **4g** (Ar = 2,4-dinitrophenyl), **4j** (Ar = 4-pyridyl), **4k** (Ar = 2-pyridyl), and **4m** (Ar = 4-methyl phenyl) also displayed significant anti-inflammatory activity. Two compounds, **4a** (Ar = phenyl) and **4d** (Ar = 4-bromophenyl) displayed negligible anti-inflammatory activity. All other compounds displayed moderate anti-inflammatory activity. Further, the number and position of substituents also count the variation in anti-inflammatory activity. Nitrogen bearing compounds **4g** (Ar = 2,4-dinitrophenyl) and **4l** (Ar = 4-nitrophenyl) showed highest anti-inflammatory activity. When the chloro substituent present on ortho-position (**4f**) of the phenyl ring displayed almost double activity as compared to a compound bearing para chloro compound (**4c**). Similarly, the difference in anti-inflammatory activity was found in compounds **4j** and **4k**, as well as **4m** and **4n** due to different arrangements of substituents on the phenyl ring.

#### 2.2.2. Analgesic Activity

Compounds under investigation showed analgesic activity ranging from 7.96% to 69.36% with a reference drug of 73.61% (Table 2).

All the tested compounds and standard drugs are evaluated at 10 mg/kg oral dose. It was identified that compound (**4l**) which showed maximum anti-inflammatory activity produces the least analgesic activity, but some selected compounds such as **4f**, **4i**, and **4n** displayed analgesic activity in a similar fashion as the anti-inflammatory activity (Table 1 and Table 2). Compound (**4k**) which exhibited the least analgesic activity was among the top-ranked anti-inflammatory activity. On the contrary, many compounds exhibiting good analgesic properties were not displayed as good anti-inflammatory activity and vice-versa (Table 1 and Table 2).

After a close understanding of anti-inflammatory and analgesic potentials of compounds under the present series, we have made a structure-activity relationship. Compounds possessing a substituted phenyl ring showed better anti-inflammatory and analgesic activity than a compound having an unsubstituted phenyl ring. In most of the cases, the substitution of electron-withdrawing groups at C-2 and C-4 positions of the phenyl ring resulted in potent compounds except compound **4d** (Ar = 4-bromophenyl) that showed negligible anti-inflammatory activity. Compound **4i** possessing two electron-withdrawing groups exhibited moderate anti-inflammatory activity but good analgesic activity. Compound (**4m**) having an electron releasing group (-CH_3_) at C-4 position exhibited better anti-inflammatory activity but less analgesic activity. On the other hand, a methyl group at C-2 showed better anti-inflammatory and analgesic agents (**4n**). A steep decrease in analgesic activity was observed when the phenyl ring was replaced by a triazole ring (**4h**).

#### 2.2.3. Acute Ulcerogenicity

Four compounds, namely, **4c** (Ar = 4-chlorophenyl), **4f** (Ar = 2-chlorophenyl), **4i** (Ar = 4-fluoro-3-chlorophenyl), and **4n** (Ar = 2-methylphenyl) were selected for their ulcerogenic activity. This selection was based on their anti-inflammatory and analgesic activity. Compounds were evaluated at an oral dose of 30 mg/kg relative to 10 mg/kg indomethacin.

The ulcerogenic activity of these compounds revealed that all the compounds showed a lesser severity index for ulcerogenicity than indomethacin (Table 3). Compound **4n** exhibited the highest severity index of 0.833 but it was only 20% of the severity shown by the standard. Mainly, compounds **4f**, **4i**, and **4n** displayed excellent anti-inflammatory, an analgesic with reduced ulcerogenic potential. Significant reduction in ulcerogenecity is ranging from 0.500 ± 0.129 to 0.833 ± 0.210, whereas standard drug indomethacin showed a high severity index of 4.500 ± 0.316.

#### 2.2.4. Lipid Peroxidation

Gastrointestinal (GI) ulceration, bleeding, and renal problems are common complications of NSAID’S consumption, which is directly related to lipid peroxidation. It has been evidenced that drugs having less ulcerogenecity showed reduced malondialdehyde (a byproduct of lipid peroxidation) content [4,41]. We have examined the lipid peroxidation (LP) of compounds which exhibited maximum anti-inflammatory and analgesic activities (**4c**, **4f**, **4i**, **4n**). It was measured as the nmol of MDA/100 mg of gastric tissue. We have found the Lipid peroxidation value was maximum 6.71 ± 0.18 for indomethacin, whereas 3.89 ± 0.17, 4.08 ± 0.22, 4.26 ± 0.12, and 4.81 ± 0.13 for compounds **4i**, **4c**, **4f**, and **4n**, respectively. It was interesting to mention that all these compounds having electron-withdrawing functionality on the phenyl ring (except **4n**) exhibited less ulcerogenecity with reduced lipid peroxidation (Table 3).

#### 2.2.5. DFT Results

**Figure 2:**
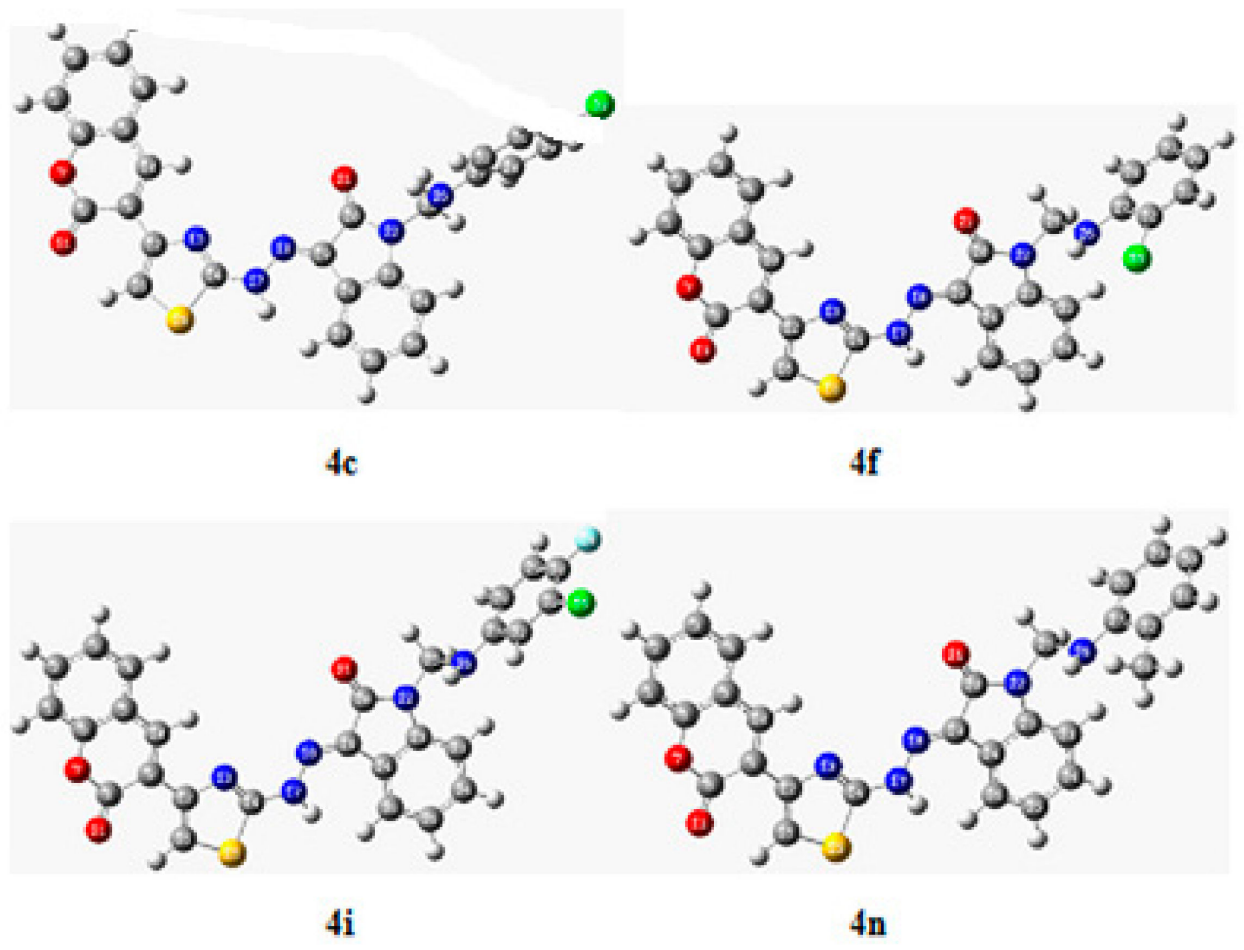
Optimized structure with the numbering of In-H synthesized derivatives.

As mentioned above, only the synthesized derivatives (In-H) that exhibited maximum anti-inflammatory and analgesic activities derivatives are subjected to the lipid peroxidation (LP) test (Table 4, Figure 2). The tested In-H derivatives show the ability to scavenge LOO• free radicals. To shed light on the small observed lipid peroxidation inhibition of In-H derivatives, bond dissociation enthalpies of the of i-NH function groups and ionization potential energies of the tested compounds were calculated at the B3P86/6-311+G(d,p) level of theory (Table 3 and Table 4).

The tested compounds showed similar lipid peroxidation with a small variation between their values. This result is confirmed by the small differences of BDEs of the active 17-NH group and IP energies, where the maximum variations of BDEs and IPs are 0.03 kcal/mol and 0.08 eV, respectively.

#### 2.2.6. In Silico Study

##### Target Protein Selection and Retrieval 

The 3D structure of target protein COX-2 from two different organisms, i.e., mouse and human were retrieved from the protein data bank having PDB id 3NT1 and 5F91, respectively [42,43].

##### Protein (COX-2) Preparation and Validation 

**Figure 3:**
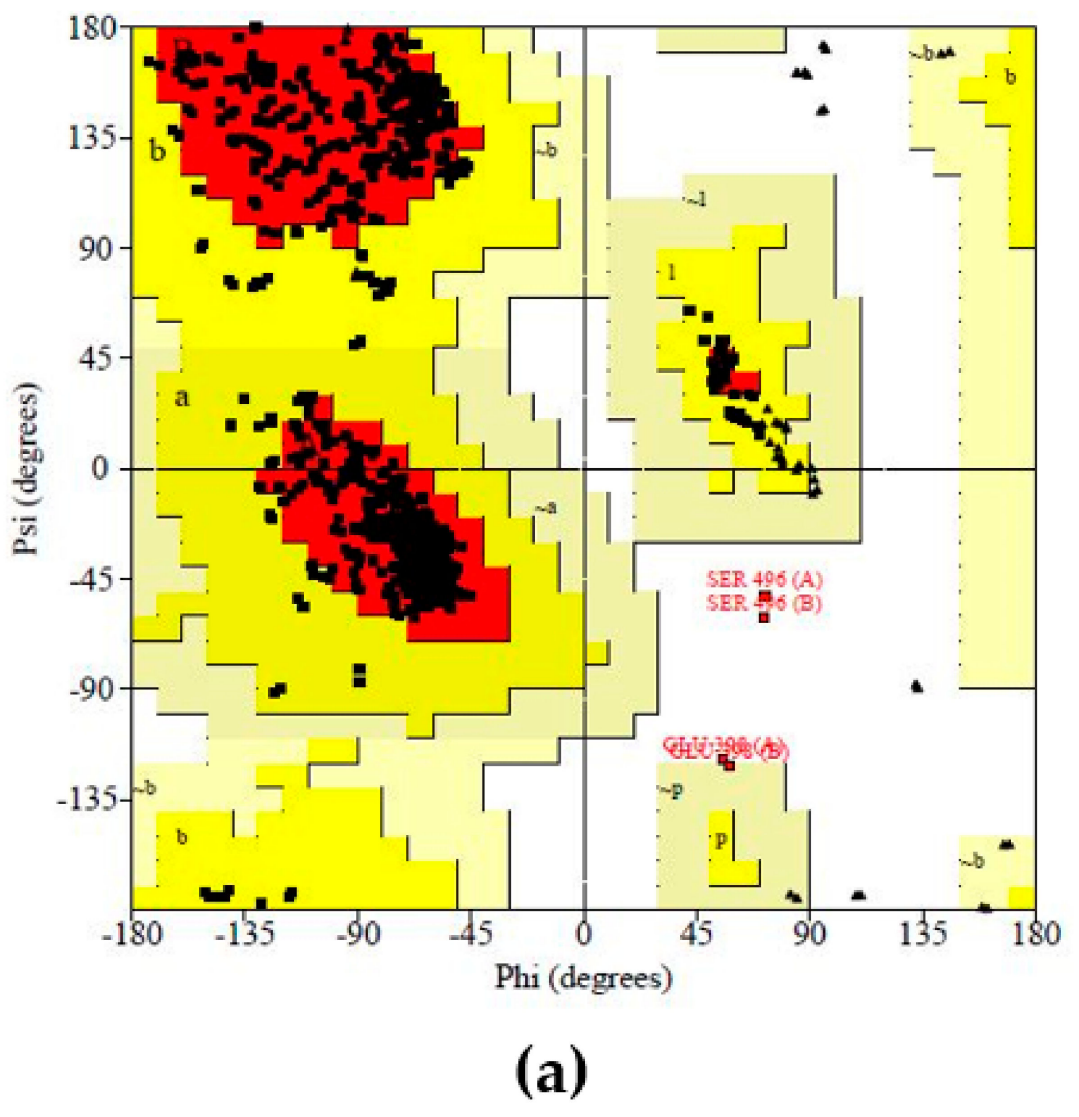

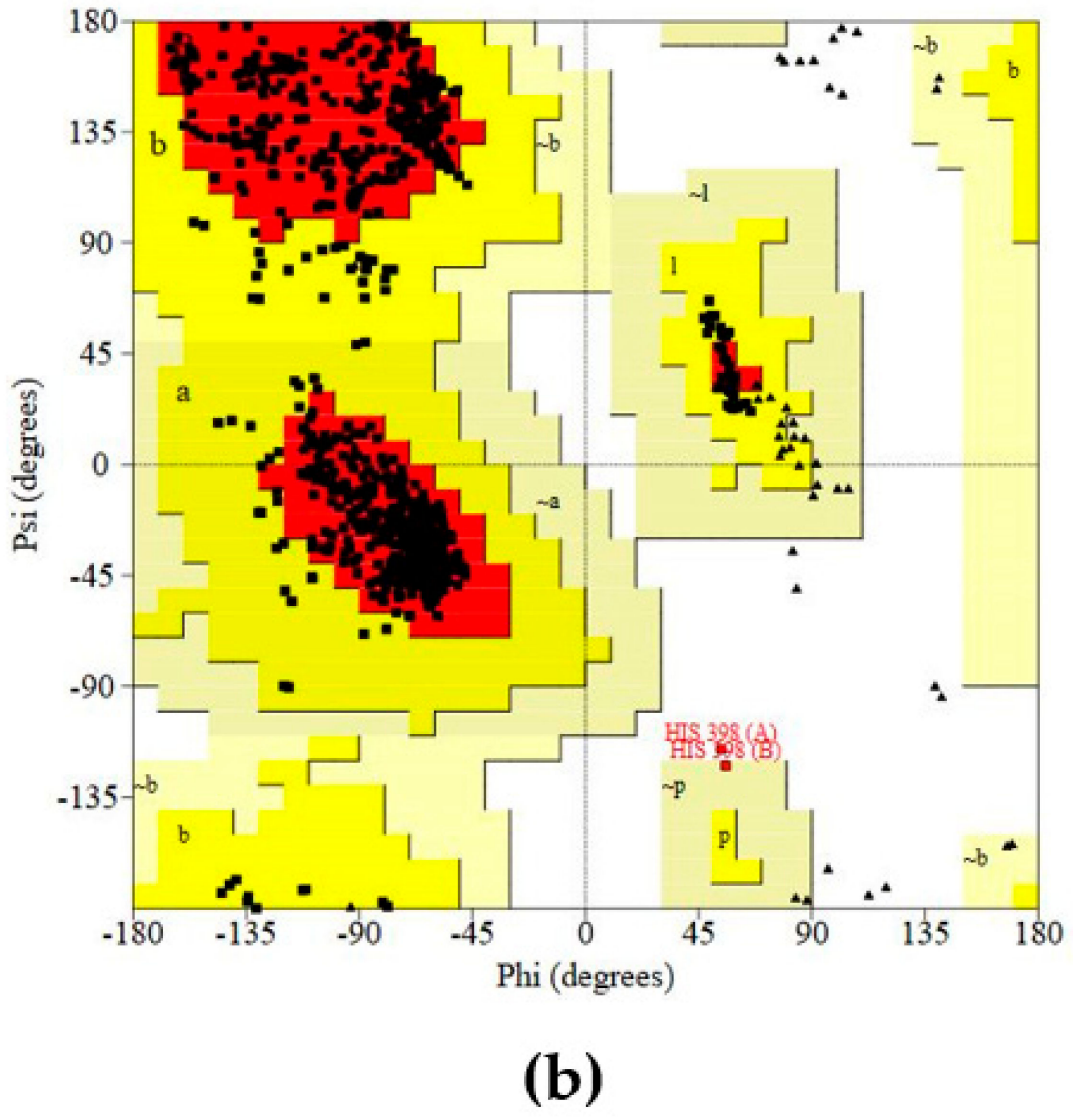
Ramachandran plot validating the prepared protein structure of cyclooxygenase (COX-2) from (**a**) mouse (PDB ID 3NT1) (**b**) human (PDB ID: 5F19).

The protein structures obtained from PDB were modified suitably for the molecular docking studies. The modified protein structures were then validated through the Ramachandran plot. The Ramachandran plot of these two-target proteins is shown in Figure 3a,b.

The evaluation of phi/psi angles validates the prepared protein structures as most of the residues are in the most favored region. In the case of 3NT1, 90.8% amino acid residues are in the most favored regions whereas 8.8%, 0.1%, and 0.3% are in additional allowed regions, generously allowed regions, and disallowed regions, respectively. Similarly, in 5F91 90.7%, 9.1%, 0.1%, and 0.1% amino acid residues are in the most favored regions, additionally allowed regions, generously allowed regions, and disallowed regions, respectively. The result obtained allows the use of these structures for further molecular docking studies.

##### Prediction and Evaluation of the Binding Site in COX-2

**Figure 4:**
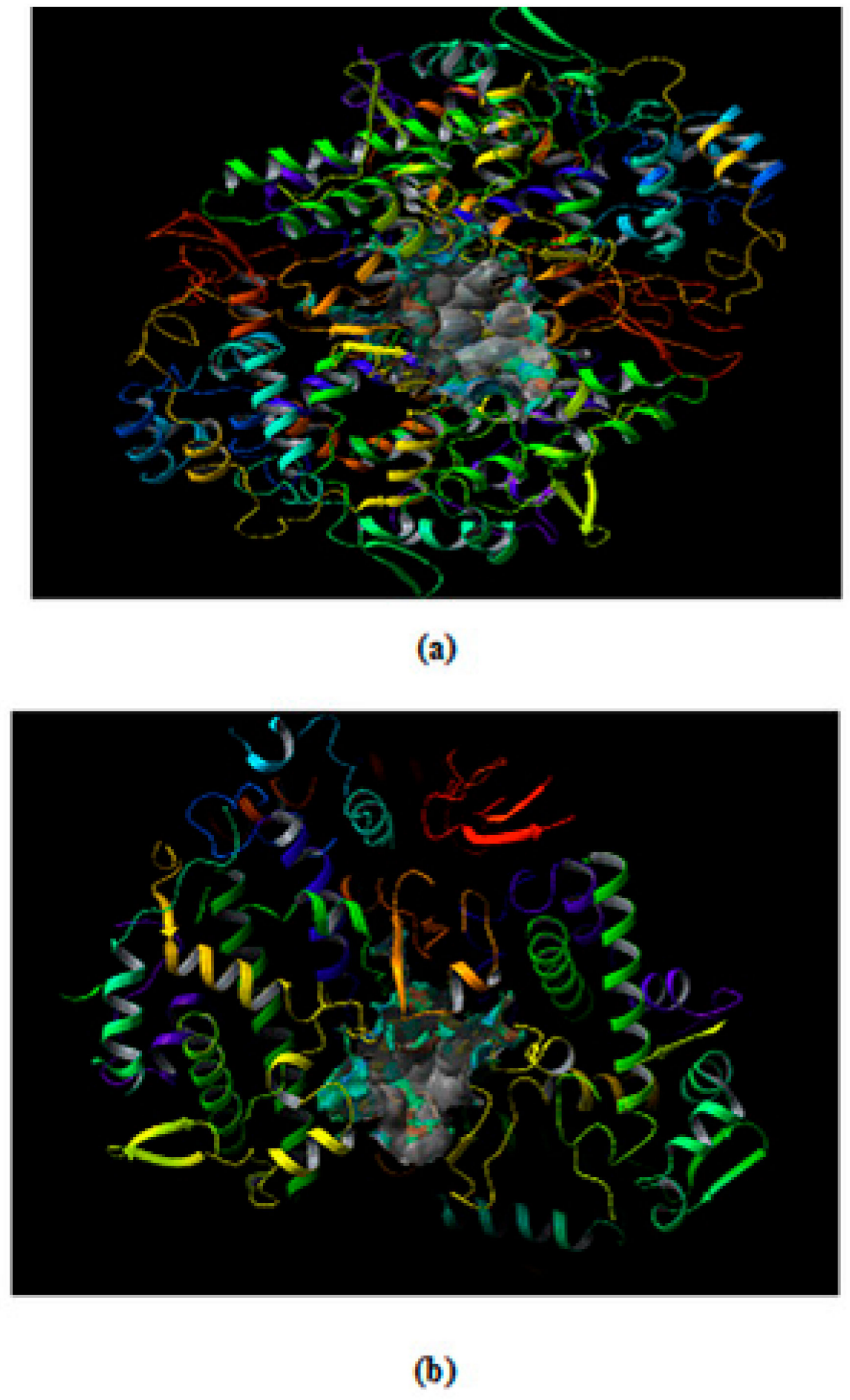
Predicted binding site in COX-2 (target protein) from (**a**) mouse (PDB ID 3NT1) (**b**) human (PDB ID: 5F19).

The site map application predicts five different drug binding sites in both the target proteins. The site score for the protein 3NT1 was 1.078, 1.053, 1.048, 1.034, and 0.991. Similarly, the site score obtained for the protein 5F19 was 1.082, 1.051, 1.046, 1.034, and 0.990. As a rule of thumb, the binding sites having a score above one are considered as druggable pockets. In the present in silico study, the site with the highest score was selected for the molecular docking studies. The druggable pocket inside the respective target proteins is shown in Figure 4a,b.

##### Ligand Preparation 

The lowest energy conformation of each test ligand (**4a**–**4n**) was prepared for the docking studies as per the standard guidelines and used in the molecular docking studies.

##### Grid Generation in the Target Protein COX-2

After the determination of the exact location of the drug binding site in each target, the protein grid was generated around the binding sites to specify the volume and location of the druggable pocket.

##### Molecular Docking Studies 

All the synthesized ligands (**4a**–**n**), i.e., the derivatives of 1-(substituted phenyl aminomethyl)-3-(2-(4-(2-oxochroman-3-yl) thiazol-2-yl) hydrazono) indolin-2-ones (**4a**–**n**) were docked inside the binding pocket of target proteins COX-2 (PDB ID: 3NT1 and 5F19) using Glide application (version 7.0, Schrödinger, New York, USA). To compare the docking efficacy of test ligands, indomethacin was used as a reference/control drug. The molecular docking results were evaluated on the basis of docking score, E-model score, and binding energy as summarized in Table 5. 

**Figure 5:**
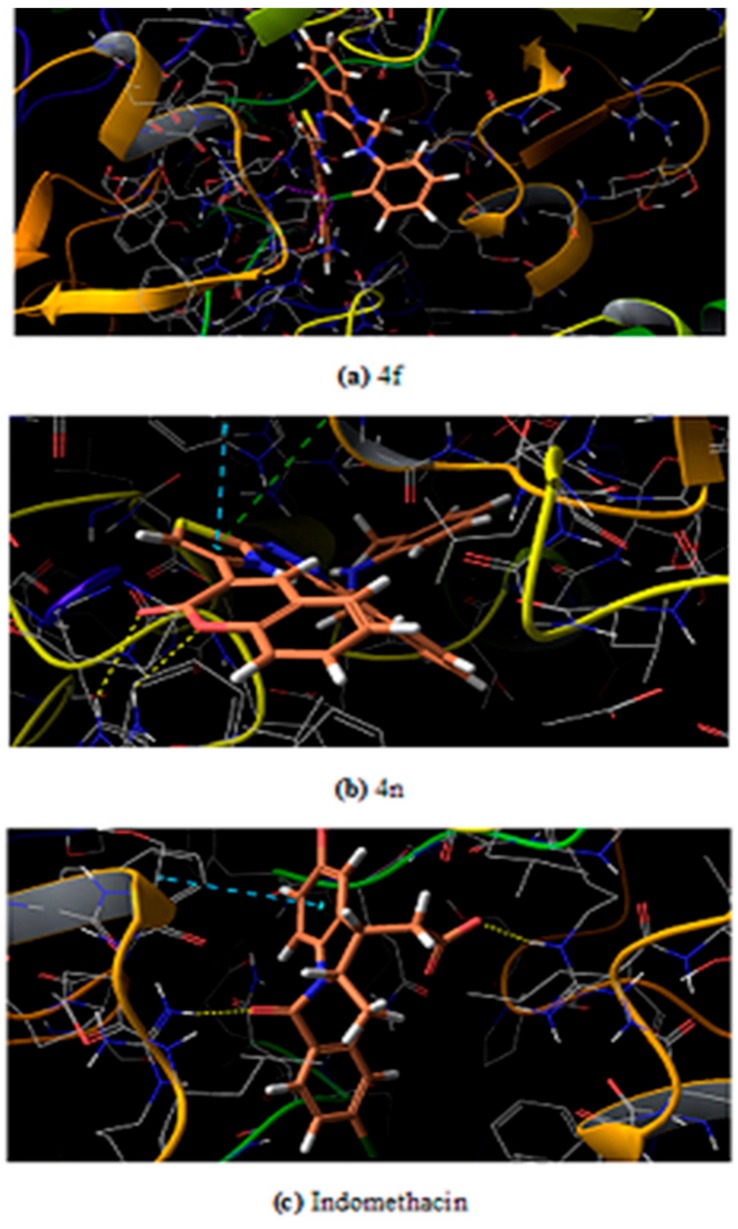
Test ligands and control drug (**a**) **4f**, (**b**) **4n**, (**c**) Indomethacin, docked inside the binding pocket of COX-2 from a mouse.

**Figure 6:**
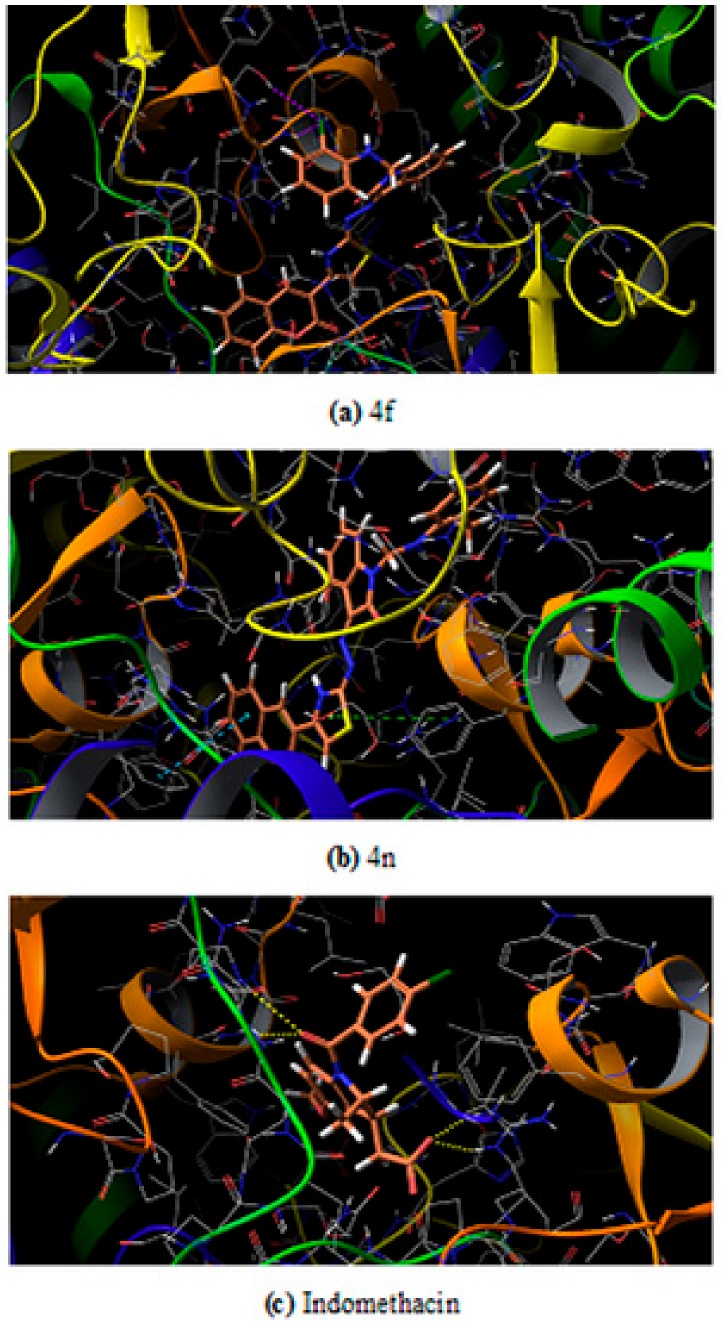
Test ligands and control drug (**a**) **4f**, (**b**) **4n**, (**c**) Indomethacin, docked inside the binding pocket of COX-2 from the human.

The maximum test ligands that are **4 a**–**c**, **e**, **g**–**n** showed docking score lower than the control/reference drug (−6.324 kcal/mol) against the mouse target protein. A similar pattern of docking score is observed against human target protein where all test ligands (**4a**–**n**) have lower docking scores as compared to control (docking score −6.109 kcal/mol). It was believed that a low binding energy dock conformer exhibited maximum stability. The two best compounds on the basis of experimental results is **4n** and **4f**. **4n** and **4f** have docking score −7.077 and −6.859 kcal/mol against human target protein, respectively. The same two ligand **4n** and **4f** have a score −6.693 and −6.071 kcal/mol against mouse target protein, respectively. The docked ligands (**4n** and **4f**) inside the binding pocket of the respective target proteins (3NT1 and 5F19) is shown in Figure 5 and Figure 6.

The further efficacy of the docking is interpreted in terms of interaction that exists between the ligand and the surrounding amino acid residues inside the druggable pocket. The overall binding interaction (in terms of bonding) for each ligand is summarized in Table 6 and Table 7 for the proteins 3NT1 and 5F19, respectively.

**Figure 7:**
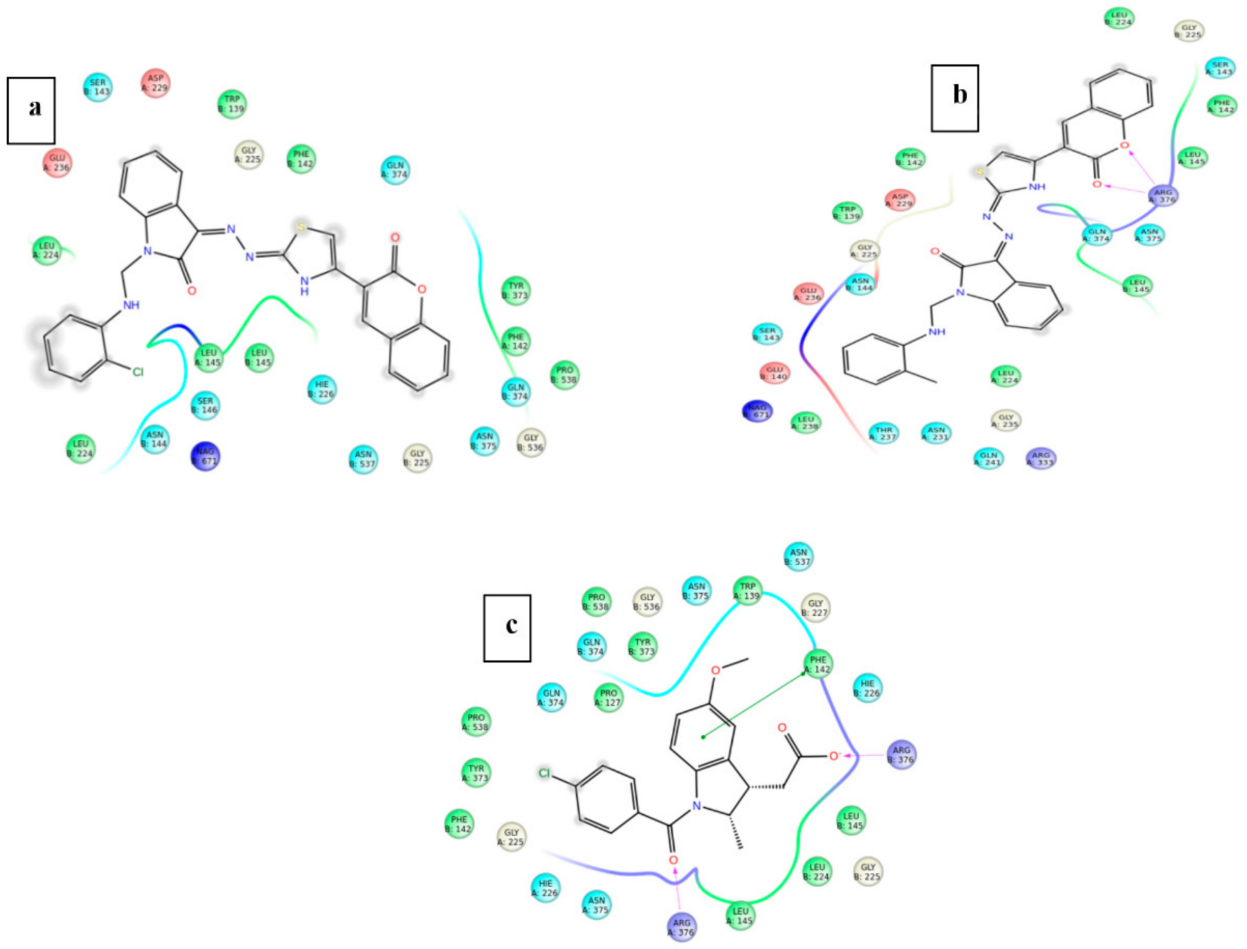
Ligand interaction diagram of test ligand/control drug (**a**) **4f**, (**b**) **4n**, (**c**) Indomethacin, with the target protein COX-2 from the mouse.

**Figure 8:**
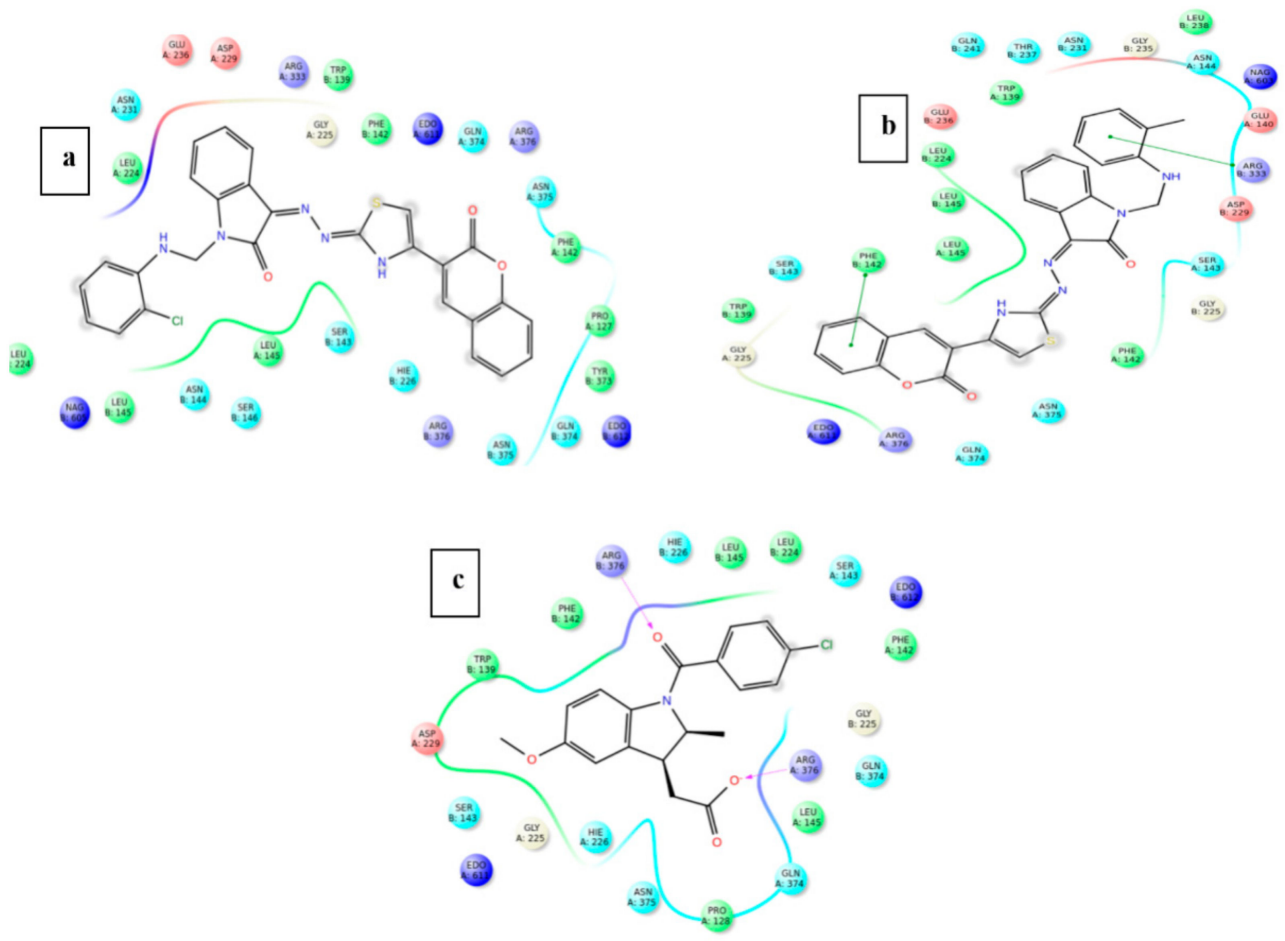
Ligand interaction diagram of test ligand/control drug (**a**) **4f**, (**b**) **4n**, (**c**) Indomethacin, with the target protein COX-2 from the human.

Among the two potent ligands, **4n** is more suitable for drug candidates as it possesses a strong affinity towards the target proteins. In 3NT1, it forms two hydrogen bonds with Arg 376, whereas in 5F19 two pi–pi stacking exists with the involvement of Phe 142 and Arg 333. In the case of **4f**, there is no hydrogen bonding or pi–pi interaction is observed (whether it is 3NT1 or 5F19). All these interactions are shown in Figure 7 and Figure 8 as a ligand interaction diagram. The molecular docking studies suggest that these two derivatives had potential to be used as a NSAID drug as it has the ability to block the COX-2 enzyme.

##### ADME Profiling

The suitability of test ligands as drug candidates depending on their pharmacokinetic behavior was also assessed using the in silico approach. Many drug candidates fail at a later stage of a clinical trial due to their poor pharmacokinetic performance. In order to avoid such failure and save time, energy, and money, in silico ADME profiling is a good choice [44]. The result of in silico ADME profiling is presented in Table 8. The result obtained suggests that the values of test parameters are within the recommended range and can be used as a drug candidates (https://www.mdpi.com/2504-3900/41/1/8). 

The oral drug absorption is predicted in terms of apparent Caco-2 permeability (QPPlogCaco) that represents the gut-blood barrier. The value above 500 indicates a great absorption while below 25 is considered a poor score [44]. The ligand **4n** and **4f** have QPPlogCaco value 619.284 and 479.473 that is very good as compared to indomethacin that has a score of 185.783 only. The Madin–Darby canine kidney (MDCK) cell model is used to investigate the apparent MDCK cell permeability [45]. The score above 500 is considerably good that is obtained in the case of both the potent test ligands. The apparent MDCK permeability score for **4n** and **4f** is 586.303 and 809.359 whereas the standard drug has a value of 251.855. The percent human abortion of both the potential ligand is also comparable to the standard and above 80%. The test ligands are also found to be obeying the Lipinski rule of 5.

##### Statistical Analysis

Data used in the experimental pharmacological section was used as the mean ± standard error (SEM). One way analysis of variance (ANOVA) and Dennett’s multiple comparison test techniques was employed to compare between the test, control, and standard group, utilizing statistical software GraphPad Prism version 5.00, California corporation, San Diego, USA. Such results showed significantly different at *p* < 0.05.

## 3. Materials and Methods 

Melting points were evaluated in open capillary tubes and are uncorrected. 5 PC FTIR spectrometer (Thermo scientific, New York, NY, USA, Bruker DRX-300 FT NMR, Bruker, Karlsruhe, Germany) spectrophotometer and Jeol-JMS-D-300 mass spectrometer (70 eV) (Jeol, Tokyo, Japan) for IR, NMR, and mass, respectively were used to characterize the compounds. 

### 3.1. Chemistry

#### 3.1.1. Preparation of 2-(2-Oxoindolin-3-Ylidene)Hydrazine Carbothioamide (2)

A combination of isatin (0.01 mole) and thiosemicarbazide (0.01 mole) was placed in a 100 mL round bottom flask with 50 mL of methanol as solvent, refluxed for 2 h, and then put onto the ice. The obtained was filtered, dried, and recrystallized using methanol.

#### 3.1.2. Preparation of Deep Eutectic Solvent (DES)

A mole ratio (1:2) of choline chloride and urea were chosen to prepare DES as per the reported method [28].

#### 3.1.3. Preparation of 3-(2-(4-(2-Oxochroman-3-Yl) Thiazol-2-yl) Hydrazono) Indolin-2-One Using Deep Eutectic Solvent and Ultrasound (3)

In a specially designed sonicating flask, an equimolar (0.01 mole) quantity of 3-bromoacetyl coumarin and 2-(2-oxoindolin-3-ylidene) hydrazinecarbothioamide (2) with 8 g of prepared DES was added. A sonicating probe of 26 kHz frequency at 40% amplitude was submerged into the reaction vessel. The completion of the reaction was monitored by taking TLC at regular intervals. Upon completion, it was poured onto crushed ice. Then, upon completion of the reaction, it was extracted by dichloromethane using a separating funnel. The organic solvent layer was collected and evaporated to get the desired product. DES was isolated and kept for future use.

#### 3.1.4. Preparation of 1-(substitutedphenylaminomethyl)-3-(2-(4-(2-Oxochroman-3-yl) Thiazol-2-yl)ydrazono)Indolin-One (4a–n)

**Scheme 2:**
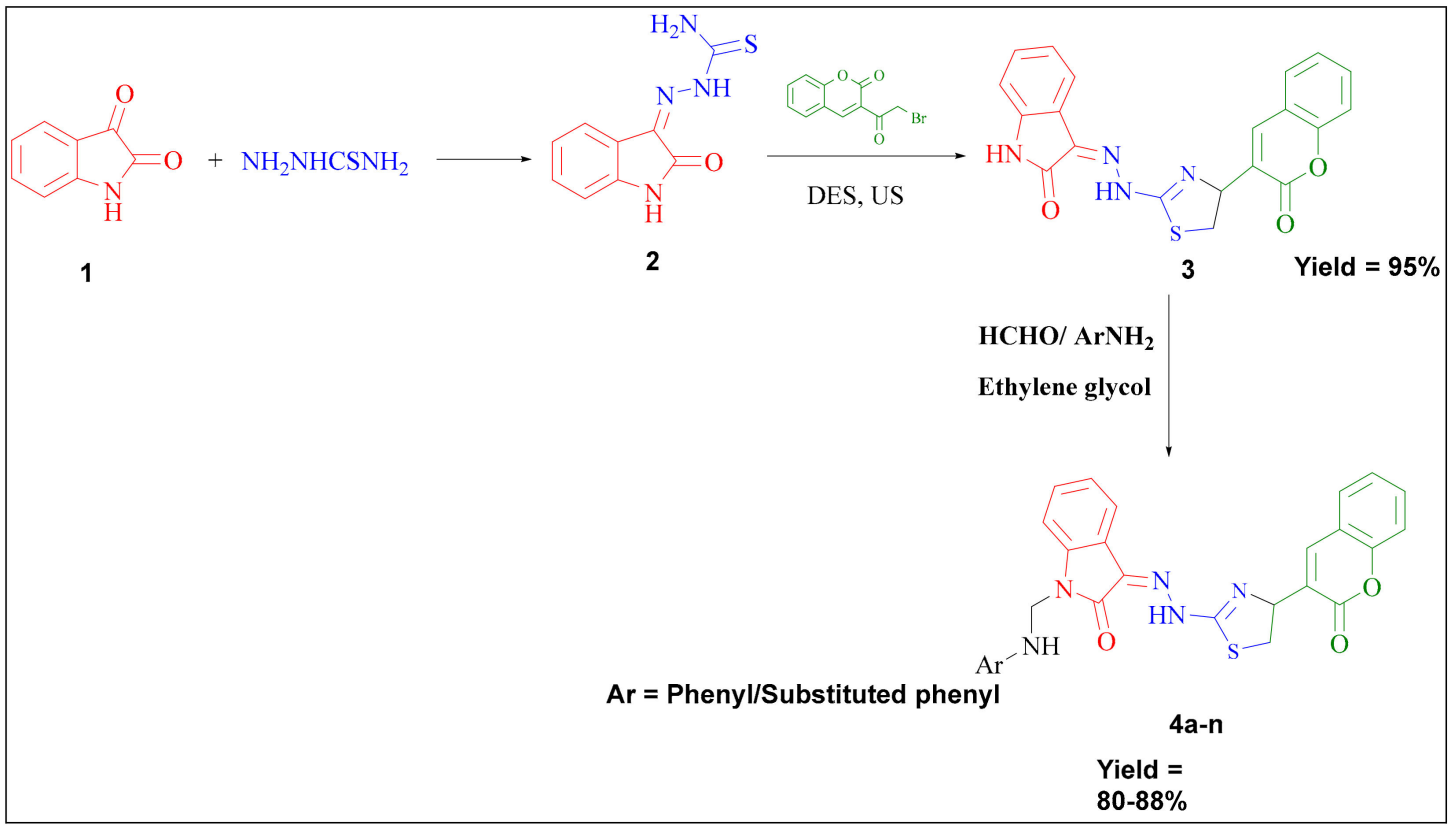
Schematic representation of the synthesis of compounds (**4a**–**n**) via key intermediate (3) isolated from deep eutectic solvent and ultrasound blend of technique.

A mixture of 3-(2-(4-(2-oxochroman-3-yl) thiazol-2-yl) hydrazono)indolin-2-one (3) (0.01 mole), substituted aromatic amines (0.01 mole) and formaldehyde (0.02 moles) in 30 ml of ethylene glycol was refluxed from 1 to 3 h. The reaction mixture was transferred onto the crushed ice upon completion, as confirmed by TLC. The solid was decanted, filtered, washed with water, dried, and recrystallized from dioxane to get affored compounds Scheme 2. 

### 3.2. Biology

#### 3.2.1. Preparation of 2-(2-Oxoindolin-3-Ylidene) Hydrazine Carbothioamide (2)

Compounds produced were assessed for their anti-inflammatory activity using the carrageenan-induced hind paw edema method [45]. The anti-inflammatory activity was carried out using Wistar albino rats of either sex (150–220 g) using a Digital Plethysmometer (Model No. 7140, UGO BASILE). The edema was induced by using 1% carrageenan solution. Indomethacin was used as a standard drug. The anti-inflammatory activity of the standard drug and tested compounds was determined at a dose of 10 mg/kg body weight. The animals were divided into groups containing six animals each and the initial paw volume of each rat was noted by the NaCl displacement method. One group was kept as control, one as standard, and the rest of the groups of compounds were to be tested. To the control group, 1% CMC solution was administered p.o. To the standard group, the standard drug was administered orally. To the test group, tested compounds were administered orally. After 60 min of the 1% CMC solution/standard drug/test compound administration, 0.1 ml of 1% (*w*/*v*) carrageenan was injected in the plantar region of the hind limb (right) of all the rats in each group including the control group. The paw volume was again measured after the time interval of 2 and 4 h. Using the following formula, inflammation was calculated as percentage inhibition for the test and reference compounds

(Final foot volume of control—final foot volume of std./test) × 100 / final foot volume of control)

#### 3.2.2. Analgesic Activity

The analgesic activity of the tested compounds was carried out by acetic acid-induced writhing method as given in the literature [9] using Swiss albino mice of either sex (25–35 g). The writh were induced in the albino mice using an intraperitoneal injection of 1% acetic acid solution. The standard drug indomethacin and test compounds were evaluated at a concentration of 10 mg/kg of the body weight. The animals were divided into groups and each group consisted of six animals. One group was kept as control, one as standard, and other as test groups. To the control group, 0.1% CMC solution was administered p.o; to the standard group, the standard drug was administered orally, and to the test group, test compounds were administered orally. After 60 min of the 0.1% CMC solution/standard drug/tested compound administration, 0.1 ml of 1% (*v*/*v*) acetic acid solution in distilled water was injected intraperitoneally in all the mice in each group including the control group. The writhing (a contraction of the abdomen, turning of trunk, and extension of hind limbs) was counted after 5 min of acetic acid administration and were counted for a period of 15 min. The percentage of analgesic activity was calculated using the following formula. 

(Mean wriths of control—mean wriths of std./test) / mean wriths of control) × 100

#### 3.2.3. Acute Ulcerogenic Activity

Acute ulcerogenic activity evaluation of the synthesized compounds was carried out according to the method described [46] using Wistar rats of either sex (180–220 g). The animals were distributed into control, group, and test group. Each group consisted of six rats. All the rats fasted for 24 h with free access to water. To the control group, 1% CMC solution was administered p.o; to the standard group, indomethacin at a concentration of 20 mg/kg was administered orally; and to the test groups, tested compounds were administered orally at a concentration of 30 mg/kg. After the dose administration animals were kept for 17 h. After this, the animals were sacrificed for the appraisal of ulcerogenic assessment. The stomach was taken out from the animal body and washed with flushing water, then with a cotton swab wetted with saline (0.9%) and pinned on wax-coated try. The glandular portion of the stomach was cleaned again with saline to closely identify the presence of a type of ulcers or hemorrhage mark using a magnifying glass. The mucosal injury of the stomach was evaluated as per the following system: 0.5 = redness; 1 = spot ulcer; 1.5 = hemorrhage streak; 2 = ulcers < 3; 3 = ulcers > 3 < 5. The value obtained as a result of the mean score of the individual treated group—mean score of control is referred to as the severity index of the gastric mucosal damage.

#### 3.2.4. Lipid Peroxidation Study

The method adopted for lipid peroxidation is the same as Ohkawaet al. [47] and the recent work of our researchers [9].

#### 3.2.5. Theoretical Details

It is well known that almost all phenolic compounds may inhibit the lipid peroxidation process due to their ability to scavenge the chain-carrying lipid peroxyl radicals, LOO•. The lipid, LH, peroxidation process is represented by three main steps initiation, propagation, and terminations. The scavenging of LOO• by the synthesized indolin-2-Ones derivatives (In-H) may refer to hydrogen atoms transferred or an electron transfer from the former to the lipid peroxyl radical. The hydrogen atom transfer is represented by the following reaction:

In-H + LOO• → In• + LOOH(1)

The above lipid peroxidation inhibition is governed by bond dissociation enthalpies (BDE) of i-NH groups of the synthesized indolin-2-ones derivatives (In-H). BDE is calculated using the following equation:

BDE = [H (In•, 298K) + H (H•, 298K)] – H (In-H, 298K)

where H is the enthalpy considered as temperature-dependent corrections [zero point energy (ZPE), vibrational, rotational, and translational energies at 298 K; H (In•, 298 K); and H (An-H, 298 K) are the enthalpies of In-H derivatives and its corresponding radical obtained after the homolytic bond dissociation of i-NH groups, respectively. H (H•, 298 K) is the enthalpy of hydrogen radical. The minimum value of BDE indicates that hemolytic bond dissociation is much easier, which is helpful in the lipid peroxidation process.

Previously, we showed the success of the hybrid functional B3B86 in rationalizing the scavenging of free radicals by synthesized natural polyphenols [48,49,50]. Hence, we extended here the use of B3P86 to the In-H synthesized derivatives as lipid peroxyl radical inhibitors. We have already tested, the basic set effect on BDEs of hispidin and isohispidin isomers by using varieties of basic sets. The obtained BDEs showed differences lower than 0.4 kcal/mol for active sites and a slight influence on IP values [48]. Consequently, a double basis set, 6-31+G(d,p), was used in this study. The 3D geometry optimization of In-H derivatives and their corresponding radicals In• were performed at the B3P86/6-6-31+G(d,p) level of theory. The ground state minima were confirmed by vibrational frequency calculations (i.e., the absence of imaginary frequencies). All DFT chemical calculations have been performed using the mentioned methodology, as implemented in the Gaussian 09 package [51].

#### 3.2.6. In Silico Study

##### Software

All the in silico experiments of the present study were carried out using the Schrodinger Maestro interface (Maestro, version 10.5, Schrödinger, LLC, New York, NY, USA, 2016) [52,53].

##### Target Protein Selection and Retrieval 

In the present study, COX-2 is selected as the target protein because the therapeutic response of NSAID’s is generated by blocking/inhibiting this enzyme. The 3D structure of COX-2 was retrieved from Protein Data Bank (PDB, https://www.rcsb.org/). There are two different structures of this enzyme available, one from the mouse and another from the human. The two structures obtained have PDB ID 3NT1 (mouse) and 5F91 (human).

##### Protein (COX-2) Preparation and Validation of Prepared Structure

The 3D structure of target proteins obtained from PDB was prepared (for further steps) using the protein preparation wizard (version 4.3, Schrödinger, New York, NY, USA). The protein preparation is a multi-step process that includes the addition of hydrogen atoms, optimization of hydrogen bonds, and elimination of any atomic level clashes. The final step of protein preparation is energy minimization that was performed at the condition 0.3 Å of RMSD and OPLS_2005 force field [54]. The protein structures prepared were further validated through the Ramachandran plot that is based on phi/psi angles evaluation.

##### Prediction and Evaluation of the Binding Site in COX-2 

To locate the ligand-binding site in the target protein, site map application (version 3.8, Schrödinger, New York, NY, USA) was used. The potency of the predicted site is decided on the basis of the site score generated by the tool. 

The binding site effectiveness is determined by several physical parameters such as size, the degree of enclosure/exposure, hydrophobic/hydrophilic character, opportunities of hydrogen bonding, etc.

##### Ligand Preparation

The derivatives of 1-(substituted phenyl aminomethyl) -3-(2-(4-(2-oxochroman-3-yl) thiazol-2-yl)hydrazono)indolin-2-ones (**4a** to **4n**) that were synthesized chemically in the previous steps were used as ligands. The chemical structure of individual ligand was drawn and prepared using LigPrep (version 3.7, Schrödinger, New York, NY, USA). The purpose of ligand preparation is to generate the 3D structure (of each ligand) that has minimum energy conformation.

##### Grid Generation in the Target Protein COX-2

The grid was created nearby the binding site in the respective target proteins. It determines the exact position and size of the binding site in terms of receptor grids that are required for the molecular docking step. The box size taken is of dimension 20 × 20 × 20 Å and the atoms were scaled by van der Waals radii of 1.0 Å having partial atomic charge less than 0.25.

##### Docking of Ligands and COX-2

The prepared ligands were docked with the COX-2 (target protein) at the respective binding site using the Glide (version 7.0 Schrödinger, New York, NY, USA) tool. The extra precision (XP) algorithm was employed for the docking operation and output is obtained in the form of docking score. It determines a possible binding pose between the target and the ligand. It also provides information about the most favorable interactions among them [55,56,57].

##### ADME Profiling

The test ligands, i.e., the derivatives of 1-(substituted phenyl aminomethyl)-3- (2-(4-(2-oxochroman- 3-yl)thiazol-2-yl)hydrazono) indolin-2-ones (**4a** to **4n**) were assessed for their pharmacokinetic efficacy through the QikProp application (version 4.7, Schrödinger, New York, NY, USA). It predicts 51 pharmacokinetic properties but the present study includes a few important parameters that are logP (Octanol/Water), apparent Caco-2 permeability QPP Caco), brain/blood partition coefficient (QPlogBB), apparent MDCK permeability (QppMDCk), (QP%) human oral absorption %, and Lipinski rule of five violations (Rule of 5).

## 4. Conclusions

In conclusion, an improved synthesis of key intermediate through the combined use of deep eutectic solvent and ultrasound is a rational approach to enhance the yield of desired compounds via an economically viable and environmentally acceptable way. The stability of reagents with DES was verified by calculating electronic energy. Further, all the final compounds (**4a**–**n**) have been evaluated as anti-inflammatory and analgesic activities. Selected compounds were further tested for ulcerogenic and lipid peroxidation potential. Only two compounds claimed to be most potent as anti-inflammatory and analgesic molecule with the highest reduction in GI toxicity. In silico study also supports the utility of these two potent ligands as drug candidates and paves the path for future drug development studies. The active compounds showed similar lipid peroxidation activities, and this was mainly due to their closest BDEs and IP values, i.e., the active compounds have the same potency to inhibit lipid radical by a hydrogen atom transfer from the active site of titled compounds to a lipid radical.

## Figures and Tables

**Table 1:** Anti-inflammatory activity of 1-(Substituted phenyl amino methyl)-3-(2-(4-(2-oxochroman-3-yl) thiazol-2-yl) hydrazono) indolin-2-one (**4a**–**n**).

Compound	% Age Inhibition of Rat Paw Edema (Dose = 10 mgkg^−1^)		Potency
	2 H	4 H	
Indomethacin	66.34 ± 0.051	82.05 ± 0.08	1.00
**4a**	38.29 ± 0.016	5.57 ± 0.041	0.06
**4b**	59.29 ± 0.73 *	45.81 ± 0.069	0.55
**4c**	59.29 ± 0.143 *	30.17 ± 0.294	0.36
**4d**	51.92 ± 0.337	6.98 ± 0.315	0.08
**4e**	62.24 ± 0.080 **	48.60 ± 0.090 **	0.59
**4f**	48.377 ± 0.219 *	72.42 ± 0.183 *	0.88
**4g**	53.57 ± 0.160 *	77.94 ± 0.184 ***	0.94
**4h**	35.39 ± 0.273	64.69 ± 0.245	0.78
**4i**	31.268 ± 0.188	63.95 ±0.218	0.77
**4j**	53.81 ± 0.120 **	77.906 ± 0.171 **	0.94
**4k**	38.095 ± 0.214	70.75 ± 0.165	0.86
**4l**	54.76 ± 0.228 **	80.94 ± 0.149 ***	0.98
**4m**	53.27 ± 0.183 *	78.42 ±0.183 **	0.95
**4n**	42.57 ± 0.213	69.58 ± 0.133	0.84

* *p* < 0.05, ** *p* < 0.01, *** *p* < 0.001.

**Table 2:** Analgesic activity of 1-(substituted phenyl amino methyl)-3-(2-(4-(2-oxochroman-3-yl) thiazol-2-yl) hydrazono) indolin-2-one (**4a**–**n**).

Compound	Mean with ± SEM	% Analgesic Activity (Dose = 10 mgkg^−1^)	Potency
Indomethacin	8.55 ± 0.394	73.61 ± 0.315 *	1.00
**4a**	17.00 ± 0.2582	47.54 ± 0.7071 *	0.64
**4b**	24.00 ± 0.3651	25.94 ± 0.5802 **	0.35
**4c**	13.00 ± 0.2582	59.88 ± 0.8458 *	0.81
**4d**	18.50 ± 0.4282	42.91 ± 0.710 ***	0.58
**4e**	16.88 ± 0.222	47.91 ± 1.0049 *	0.65
**4f**	9.93 ± 0.386	69.36 ± 0.5845 *	0.94
**4g**	20.09 ± 0.3561	38.01 ± 1.0035 **	0.51
**4h**	23.83 ± 0.3073	26.47 ± 0.3165 *	0.35
**4i**	10.93 ± 0.3128	66.27 ± 1.0072 *	0.90
**4j**	17.13 ± 0.539	47.14 ± 0.4018 ***	0.64
**4k**	29.83 ± 0.3073	7.96 ± 0.4318 *	0.10
**4l**	17.83 ± 0.3079	44.98 ± 0.3361 *	0.61
**4m**	21.83 ± 0.2051	32.64 ± 0.8454 **	0.44
**4n**	10.00 ± 0.3651	69.14 ± 0.6892 *	0.93

* *p* < 0.05, ** *p* < 0.01, *** *p* < 0.001.

**Table 3:** Ulcerogenic activity and lipid peroxidation of 1-(substituted phenyl amino methyl)-3-(2-(4-(2- oxochroman-3-yl) thiazol-2- yl) hydrazono)indolin-2-one.

Compound	Severity Index	Nanomoles of MDA Content ± SEM/ 100 mg Tissue
Control	0.0	3.16 ± 0.12 *
Indomethacin	4.500 ± 0.316	6.71 ± 0.18 *
**4c**	0.666 ± 0.105 *	4.26 ± 0.12 *
**4f**	0.666 ± 0.105 *	4.08 ± 0.22 *
**4i**	0.500 ± 0.129	3.89 ± 0.17 *
**4n**	0.833 ± 0.210 *	4.81 ± 0.13 *

* *p* < 0.05.

**Table 4:** Bond dissociation enthalpies (BDEs) (kcal/mol) of i-NH groups of the indolin-2-ones (In-H) synthesized derivatives and its corresponding ionization potential energies calculated at the B3P86/6-31+G(d,p) level of theory.

Compound	IP (eV)	17-NH	26-NH	Lipid Peroxidation Inhibition
**4c**	−5.96	62.03	72.58	4.08 ± 0.22
**4f**	−5.97	62.08	75.60	4.26 ± 0.12
**4i**	−6.04	62.05	72.84	3.89 ± 0.17
**4n**	−5.80	62.05	72.02	4.81 ± 0.13

**Table 5:** Summary of molecular docking score of different ligands against Cox-2 (target protein) from mouse (3NT1) and human (5F19).

S. No	Ligand	Docking Score (kcal/mol)		E-Model Score (kcal/mol)		Energy (kcal/mol)	
		Mouse	Human	Mouse	Human	Mouse	Human
1	**4a**	−7.050	−6.834	−84.018	−85.694	−59.395	−60.236
2	**4b**	−8.552	−7.398	−93.570	−91.718	−61.736	−63.562
3	**4c**	−6.847	−7.368	−89.139	−90.888	−65.402	−63.532
4	**4d**	−6.271	−7.419	−86.746	−90.209	−64.531	−63.856
5	**4e**	−6.995	−7.200	−88.939	−90.453	−63.810	−64.065
6	**4f**	−6.071	−6.859	−78.327	−79.342	−58.256	−59.290
7	**4g**	−7.247	−7.426	−92.213	−92.642	−65.665	−64.682
8	**4h**	−8.422	−7.760	−99.511	−97.487	−65.199	−66.691
9	**4i**	−7.242	−7.446	−92.293	−93.023	−64.084	−64.835
10	**4j**	−8.120	−7.250	−97.069	−89.953	−64.452	−62.022
11	**4k**	−7.887	−7.261	−94.176	−90.861	−63.958	−63.245
12	**4l**	−8.447	−7.544	−95.832	−81.672	−65.289	−56.454
13	**4m**	−7.898	−6.803	−85.845	−84.328	−59.419	−60.257
14	**4n**	−6.693	−7.077	−85.842	−87.991	−62.568	−61.802
15	Indomethacin	−6.324	−6.109	−57.309	−58.132	−39.727	−40.695

**Table 6:** Type of interaction and amino acid residues involved in that interaction inside the binding pocket of the Cox-2 enzyme from mouse (4NT1).

S. No	Ligand	Types of Interaction	Interacting Residues
1	**4a**	Solvation effect	-
2	**4b**	1 H-bond, 1 pi–pi stacking	Phe 142, Asn 37
3	**4c**	1 pi–pi stacking	Phe 142
4	**4d**	1 H-bond, 1 pi–pi stacking	Trp, 139, Phe, 142
5	**4e**	2 H-bond	Leu 145, Ser 146
6	**4f**	Solvation effect	-
7	**4g**	2 H-bond	Leu 145, Ser 146
8	**4h**	1 H-bond, 1 pi–pi stacking	Phe 142, Gly 225
9	**4i**	2 pi–pi stacking	Phe 142, Arg 133
10	**4j**	3 H-bond	Glu 142, Arg 376
11	**4k**	1 pi–pi stacking	Phe 142
12	**4l**	3 H-bond, 1 pi–pi stacking	Phe 142, Val 228, Asn 375, Asn 537
13	**4m**	1 H-bond, 1 pi–pi stacking	Phe 142, Asn 375
14	**4n**	2 H-bond	Arg 376
15	Indomethacin	2 H-bond, 1 pi–pi stacking	Phe 142, Arg 376

**Table 7:** Type of interaction and amino acid residues involved in that interaction inside the binding pocket of the Cox-2 enzyme from mouse (5F19).

S. No	Ligand	Types of Interaction	Interacting Residues
1	**4a**	1 pi–pi stacking	Phe 142
2	**4b**	2 pi–pi stacking	Phe 142, Arg 333
3	**4c**	2 pi–pi stacking	Phe 142, Arg 333
4	**4d**	2 pi–pi stacking	Phe 142, Arg 333
5	**4e**	2 H-bond	Leu 145, Ser 146
6	**4f**	Solvation effect	-
7	**4g**	3 H-bonds	Leu 145, Ser 146, Nag 605
8	**4h**	2 H-bond, 1 pi–pi stacking	Arg 333, Arg 376
9	**4i**	2 pi–pi stacking	Phe 142, Arg 333
10	**4j**	2 H-bond	Glu140, Arg 376
11	**4k**	1 H-bond, pi–pi stacking	Trp 139, Phe 142, Arg 333
12	**4l**	2 H-bond, 2 pi–pi stacking	Phe 142, Gln 241, Arg 333
13	**4m**	2 pi–pi stacking	Phe 142, Arg 333
14	**4n**	2 pi–pi stacking	Phe 142, Arg 333
15	Indomethacin	2 H-bonds	Arg 376

**Table 8:** Absorption distribution metabolism and excreation (ADME) profiling of all the ligands (**4a**–**n**) synthesized to be used as a drug candidate.

S. No	Ligand	Mol. Wt.	QPlogPo/w (Octanol/ Water)	Apparent Caco-2 Permeability (QPP Caco)	Brain/Blood Partition Coefficient (QPlogBB)	Apparent MDCK Permeability (QppMDCK)	Human Oral Absorption % (QP%)	Lipinski Rule of 5 Violations (Rule of 5)
1	**4a**	493.539	5.757	574.519	−1.128	540.552	100	1
2	**4b**	511.529	5.966	521.054	−1.100	880.621	84.589	2
3	**4c**	527.984	6.226	521.126	−1.051	1201.640	86.113	2
4	**4d**	572.435	6.306	521.273	−1.043	1292.410	86.581	2
5	**4e**	538.536	4.878	85.123	−2.223	59.380	64.132	2
6	**4f**	527.984	6.047	479.473	−1.121	809.359	84.417	2
7	**4g**	583.534	4.121	10.166	−3.511	5.971	43.182	2
8	**4h**	484.491	2.102	23.583	−1.616	18.982	50.863	1
9	**4i**	545.974	6.468	578.335	−0.889	2174.980	88.336	2
10	**4j**	494.927	4.735	329.574	−1.421	3.000	100	0
11	**4k**	494.927	4.703	313.043	−1.444	296.440	100	0
12	**4l**	538.536	5.003	68.904	−2.393	54.611	50.266	3
13	**4m**	507.566	6.083	574.067	−1.164	540.037	86.024	2
14	**4n**	507.566	6.027	619.284	−1.085	586.303	86.288	2
15	Indomethacin	373.835	3.679	185.783	−0.614	251.855	89.095	0

Recommended values: Molecular weight: 130.0–725; QPlogBB: −3.0–1.2; QPPlogCaco: 25 poor, >500 great; QppMDCK: 25 poor, >500 great; QPlogPo/w: −2.0–6.5; QP%: >80% is high, <25% is poor; Rule of 5: Maximum is four.
